# Recent Advances in the Use of Plant Virus-Like Particles as Vaccines

**DOI:** 10.3390/v12030270

**Published:** 2020-02-28

**Authors:** Ina Balke, Andris Zeltins

**Affiliations:** Plant virology group, Latvian Biomedical Research and Study Centre, Ratsupites 1, LV1067 Riga, Latvia; inab@biomed.lu.lv

**Keywords:** plant virus, virus-like, vaccine platform, epitope, antigen, immune response

## Abstract

Vaccination is one of the most effective public health interventions of the 20th century. All vaccines can be classified into different types, such as vaccines against infectious diseases, anticancer vaccines and vaccines against autoimmune diseases. In recent decades, recombinant technologies have enabled the design of experimental vaccines against a wide range of diseases using plant viruses and virus-like particles as central elements to stimulate protective and long-lasting immune responses. The analysis of recent publications shows that at least 97 experimental vaccines have been constructed based on plant viruses, including 71 vaccines against infectious agents, 16 anticancer vaccines and 10 therapeutic vaccines against autoimmune disorders. Several plant viruses have already been used for the development of vaccine platforms and have been tested in human and veterinary studies, suggesting that plant virus-based vaccines will be introduced into clinical and veterinary practice in the near future.

## 1. Introduction

Vaccination is one of the most powerful public health interventions of the 20th century, preventing an estimated six million deaths a year [1]. From a public point of view, vaccination programs result in cost savings that exceed investments by 16-fold [2].

**Figure 1:**
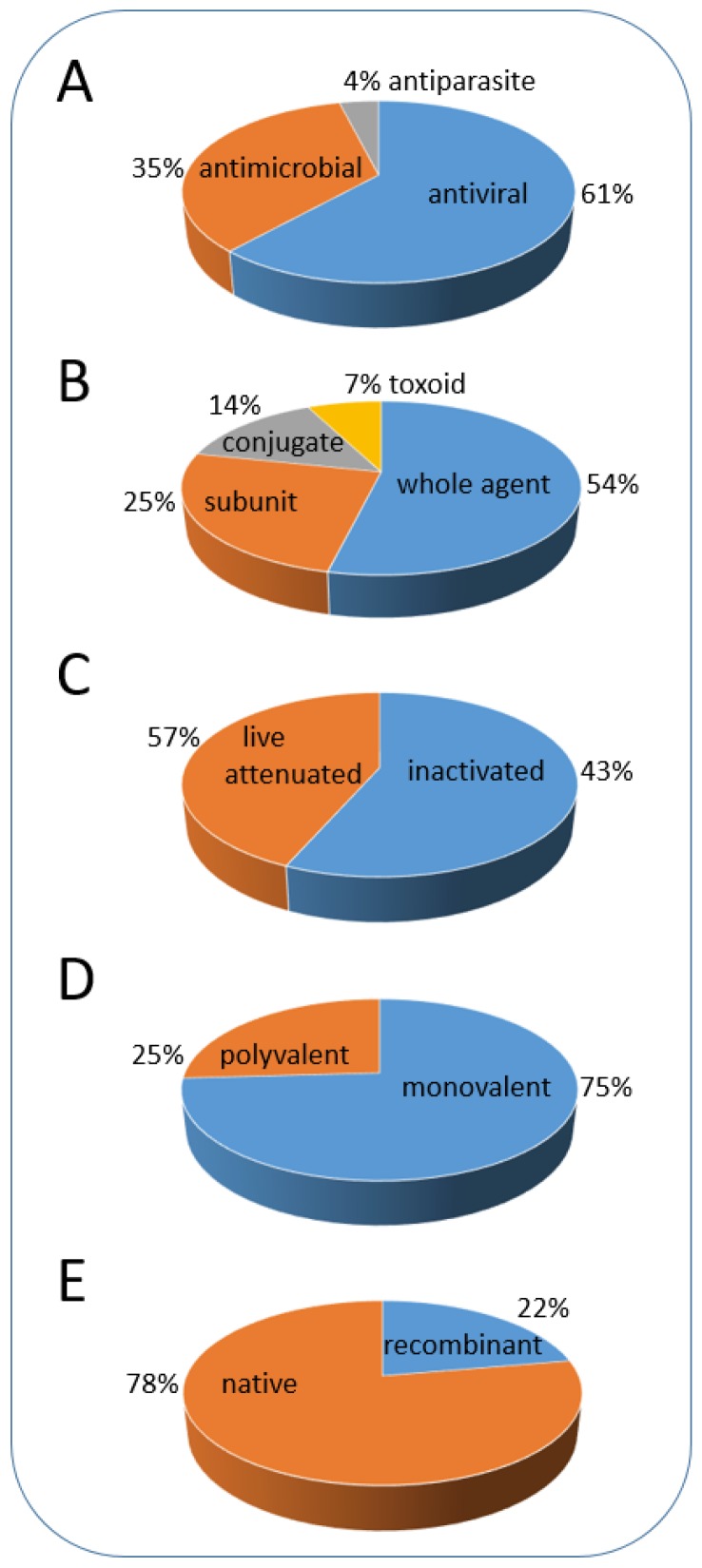
Classification of vaccines. (**A**) vaccines against different infectious agents; (**B**,**C**) vaccine types according to method of development; (**D**) vaccines containing single or multiple antigen subtypes from the same pathogen (mono-or poly-valent); (**E**) vaccines containing native or artificially generated (recombinant) antigens. The proportion (%) of a corresponding vaccine type is calculated based on the list of diseases and available vaccines (Supplementary Table S1; based on data from World Health Organization [6]).

During the last 200 years, academic scientists and the vaccine industry have developed a large number of vaccines, including approximately 50 vaccines licensed for human use and more than 300 veterinary vaccines [3]. All currently used vaccines can be classified into several types, and the classification also reflects the historical steps in vaccine development [4,5]. These types include vaccines against different infectious agents (bacteria, viruses, parasites); vaccines obtained by different methods of development; vaccines containing single or multiple antigens and recombinant vaccines (Figure 1, Supplementary Table S1). 

The very first vaccine was the smallpox vaccine introduced by Jenner more than 200 years ago [7]. He used a heterologous virus containing material originating from infected cows (cowpox) for the protection of humans against smallpox. Further development and international use of the vaccine led to the complete eradication of smallpox, which is one of the greatest achievements in medicine [4].

Targeted vaccine development began with Luis Pasteur’s discovery that pathogenic microorganisms are less virulent if cultivated under suboptimal conditions or treated with different chemicals. He introduced the concept of attenuated vaccines, which was later exploited in the generation of many vaccines, including vaccines against anthrax and rabies [4]. Later, progress in cell culture technology enabled the culture of mammalian cells and allowed the propagation of viruses, resulting in effective attenuated vaccines against polio, measles, mumps, rubella, influenza and other infectious diseases [8].

The third group of vaccines was generated by the chemical inactivation of infectious bacteria and viruses as well as bacterial toxins (toxoid vaccines). The application of increased temperatures or inactivating chemicals in the production of different vaccines is still used by industry for vaccines against cholera, polio, rabies, Japanese encephalitis and other infectious diseases [9].

The development of molecular genetics, bacteriology and biochemistry contributed significantly to the understanding of the molecular structure of living cells and allowed the identification of pathogen components potentially suitable for vaccine generation. The exploitation of this idea resulted in a long list of so-called subunit vaccines, including vaccines against *Haemophilus influenza,* meningococcus, pneumococcus (polysaccharides and their conjugates with carrier proteins) and hepatitis B (plasma-derived protein antigen) [9].

The advent of molecular biology resulted in the next breakthrough in the medicinal industry. The achievements in gene engineering have dramatically influenced the construction of vaccines. Since the 1980s, recombinant technologies have been introduced for the development of different vaccine types, such as live and attenuated recombinant bacteria and viruses, as well as the production of toxins and other protein antigens using recombinant hosts [8].

The hepatitis B (HBV) vaccine was the first subunit vaccine, and it was generated by gene engineering techniques more than 30 years ago. The expression of a cloned copy of the HBV surface antigen (HBsAg) in yeast cells resulted in the production of noninfectious virus-like particles (VLPs) [10] and allowed the replacement of a previously generated plasma-derived vaccine [11]. The success of HBV vaccines strongly stimulated the development of recombinant vaccines based on viral structural proteins and marked the beginning of a new era in rationally designed VLP platforms for the generation of prophylactic and therapeutic vaccines [12].

Using the same principle of viral coat protein (CP) expression in heterologous hosts, vaccines against cervical cancer (Gardasil and Cervarix; [13,14,15]), hepatitis A (Hecolin; [16]) and malaria (RTS,S; [17]) were constructed, clinically tested and licensed for human use in the subsequent decades. It is important to stress that the RTS,S vaccine is the first among licensed vaccines containing a VLP carrier (HBsAg) with an incorporated foreign antigen (CS).

Artificial virus-like structures derived from plant virus proteins are well known due to virus assembly studies performed since the 1950s [18]. Based on the use of carrier proteins with chemically coupled peptide antigens as promising vaccine candidates [19], Haynes et al. [20] generated an experimental vaccine using a gene engineering approach instead of chemical coupling. They combined the tobacco mosaic virus (TMV) *CP* gene with an extension encoding a C-terminally located, 8 AA-long antigenic peptide from poliovirus. The resulting VLPs purified from recombinant bacterial cells were immunogenic and stimulated the formation of antibodies against poliovirus in rats. These results, together with those of HBsAg [21], introduced the use of VLPs as a central carrier element of many experimental and licensed vaccines. Additionally, the study clearly demonstrates that nonpathogenic viruses are also suitable for vaccine generation after the introduction of relevant antigens into their structure.

Viruses and their derivatives possess several characteristics that are highly important for their use as vaccines [22]. Most likely, the most important property of viruses and VLPs is their structural organization. Structurally, viruses are constructed of hundreds or thousands of highly ordered CP molecules, which serve as repeated antigens for the mammalian immune system. These antigens on the virus surface can stimulate B cells by crosslinking B cell receptors and induce long-lasting antibody responses. In addition, most viruses have the optimal size, shape and rigidity to enter the lymphatic system through the pores in lymph vessels. This facilitates the trafficking of viral particles and VLPs to lymph nodes and their uptake by antigen-presenting cells (APCs). Moreover, viruses and VLPs encapsulate specific host-derived nucleic acids (DNA or RNA), which stimulate Toll-like receptors in APCs and serve as natural vaccine adjuvants [23,24].

**Figure 2:**
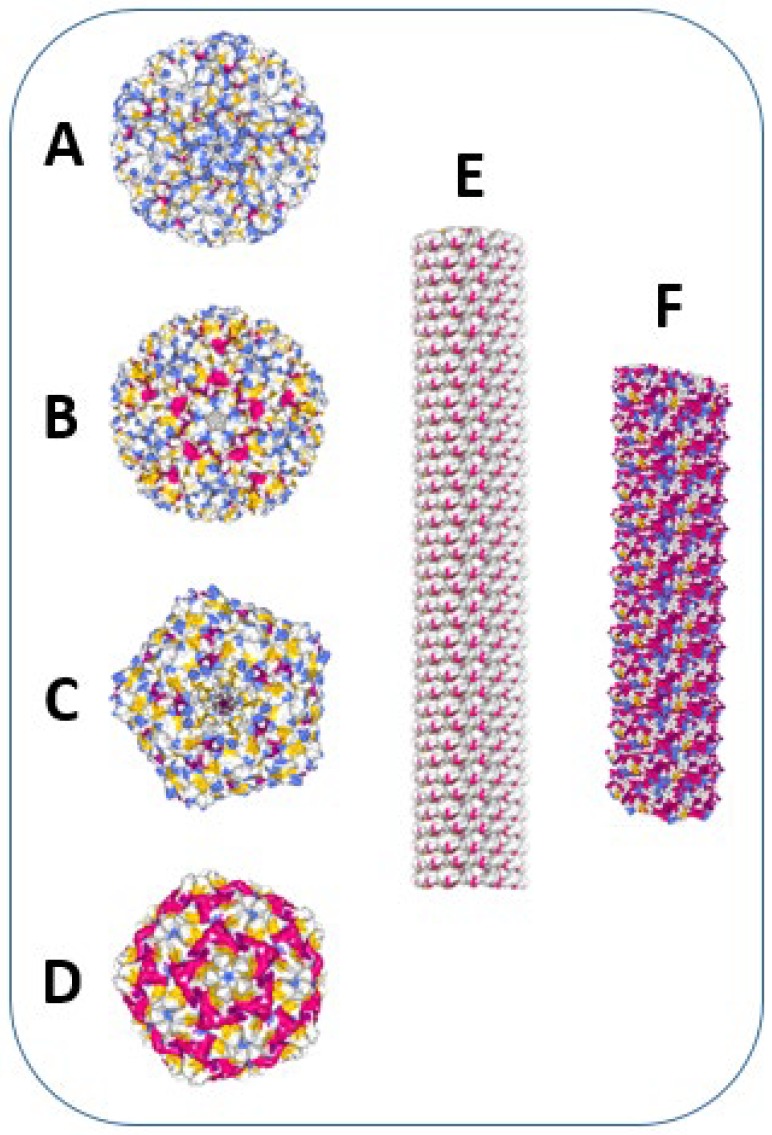
Examples of icosahedral and helical plant viruses, used for vaccine development. Images were created using Protein Data Bank and NGL 3D viewer [27]. α-helices are shown in red, β-sheets–in yellow. (**A**) Cucumber mosaic virus (CMV) structure (T = 3 symmetry, diameter = 28 nm). Image of 5OW6 [28]. (**B**) Cowpea chlorotic mottle virus (CCMV) structure (T = 3 symmetry, diameter = 29 nm). Image of 1CWP [29]. (**C**) Cowpea mosaic virus (CPMV) structure (T = 3 symmetry, diameter = 28 nm). Image of 1NY7 [30]. (**D**) Sesbania mosaic virus (SeMV) structure (T = 3 symmetry, diameter = 28 nm). Image of 1X33 [31]. (**E**) Tobacco mosaic virus (TMV) structure (cryo-EM reconstruction of a TMV fragment; particle length = 300 nm, diameter = 18 nm). Image of 3J06 [32]. (**F**) Bamboo mosaic virus (BaMV) structure (cryo-EM reconstruction of a BaMV fragment; particle length = 490 nm, diameter = 15 nm). Image of 5A2T [33].

Plant viruses and VLPs (Figure 2), compared with other VLPs, have additional advantages as vaccine carrier structures. It is well known that plant viruses are not able to infect mammalian organisms. Therefore, the probability of pre-existing immunity against plant viruses is considerably lower compared with that against VLPs derived from mammalian viruses (e.g., HBV and papilloma virus). Most plant viruses are assembled from single or a few CP molecules and demonstrate remarkable structural flexibility, allowing different manipulations, such as disassembly/reassembly, as well as chemical and genetic modifications. This enables the rational design of vaccines and the introduction of different antigens derived from infectious agents, allergens and self-molecules. Additionally, plant VLPs and other artificially generated VLPs do not contain replicating nucleic acids. This prevents the reversion of vaccines into infectious viruses, which is a serious risk factor for attenuated viral vaccines. From a technological viewpoint, plant viruses and VLPs can be produced in different recombinant hosts (bacteria, yeasts, plants, and eukaryotic cells) under cGMP conditions; the mentioned structural flexibility of plant viruses allows the construction of universal vaccine platforms [25,26].

In this review, we summarize the publicly available data on vaccines derived from plant viruses, emphasizing the newest developments in the construction of experimental vaccines. We used two databases as a source of information: the PubMed database of the US National Institutes of Health (https://www.ncbi.nlm.nih.gov/pubmed/) and the Web of Science (https://apps.webofknowledge.com). Our literature search demonstrates the rapidly growing interest in plant viruses as vaccine carriers; for example, a cumulative search for the term ‘‘plant virus vaccine’’ in Web of Science yielded 787 publication records through 2009, more than 1400 through 2014, and at least 2204 publications through the end of 2019. As revealed by the search, plant virus derivatives serve as components for at least 71 experimental vaccines against infectious diseases, 16 anti-cancer vaccines and 10 vaccines against allergies and autoimmune diseases (examples and literature citations are summarized in Supplemental Tables S2, S3 and S4). The increasing interest and recent publications stimulated us to summarize and discuss the latest developments in the design of vaccines based on plant viruses. Descriptions of other plant VLP applications can be found in several recent review articles [34,35,36,37,38].

## 2. Plant VLP-Derived Vaccines against Infectious Diseases

The efforts of the vaccine industry have resulted in a comparably long list of licensed prophylactic vaccines that effectively prevent infection by different infectious agents (Supplementary Table S1). However, the efficacy of current vaccines is moderate or even poor in some cases and does not ensure long-lasting protection and the eradication of the corresponding diseases [39]. Additionally, there is an urgent need for vaccines against viral and bacterial pathogens, such as human immunodeficiency virus, tuberculosis, malaria, and many other agents, as well as emerging diseases, such as Zika virus, Dengue, Ebola and SARS. Therefore, interest in alternative solutions for the generation of new vaccines and the improvement of existing vaccines is continuously growing.

Experimental vaccines based on plant viruses have been constructed and tested for more than 30 years since the first TMV-based vaccine was developed against poliovirus [20]. In this chapter, we will discuss the recent development of plant virus-derived prophylactic vaccines against important pathogens such as influenza and malaria.

In most cases, plant virus-derived vaccines are peptide vaccines that are genetically fused or chemically coupled to viral CP. Peptide vaccines can stimulate the formation of neutralizing antibodies and ensure protection against the corresponding pathogen in animal models (Suppl. Table S2). The advantages of this approach include the reduced influence of short peptides on the structural integrity of viral carriers and the easy production of experimental vaccines. However, the antigen is frequently not a linear AA sequence but a spatially complex structure involving different parts of the antigenic protein. For such antigens, peptide-based vaccines are not suitable for stimulating protection via the immune response. Therefore, several authors have suggested the usage of whole proteins as antigens, which can result in epitope structures closely related to native ones and elicit neutralizing antibodies after immunizations. Genetic fusions of whole antigenic proteins with viral CPs negatively influence VLP formation in most cases. However, sometimes plant viral coats are able to accommodate even very long antigenic sequences on their surfaces [40,41]. As alternatives to genetic fusions, other approaches can result in antigen presentation, such as chemical coupling or physical binding of the antigen to the VLP surface using binding partner molecules. Successful examples of the generation of vaccines using the chemical or enzymatic coupling of whole antigens include studies of experimental vaccines against *Francisella tularensis* [42], influenza [43], *Yersinia pestis* [44], *Plasmodium vivax* [45], and Zika virus [46].

Methods used for the incorporation of whole antigen sequences into viral *CPs*, allowing the preservation of viral morphology, are discussed in recent review articles [25,47].

**Figure 3:**
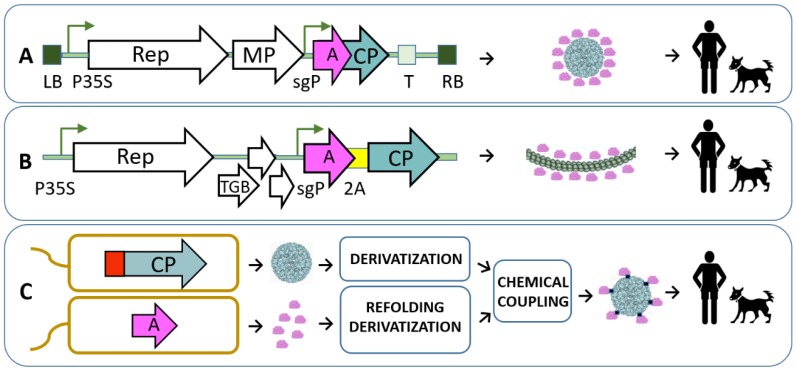
Examples of recombinant vaccine platforms used for introduction of different antigens in plant viral structures. (**A**) «launch» vector [51] based on TMV for production of antigen-containing viruses in plants. LB and RB denotes agrobacterial shuttle vector T-DNA region left and right borders; P35S—CaMV promoter; sgP—subgenomic promoter; T—transcription terminator; Rep, MP—TMV replicase and movement protein; pink and turkish arrows denote the relative position of antigen (A) and AlMV coat protein gene (*CP*), respectively [41]. (**B**) BaMV-based vector for production of antigen (A)-containing mosaic viruses in plants. *sgP*—subgenomic promoter; A—antigen-coding sequence; 2A—self-processing peptide sequence; *Rep*, *TGB*, *CP*–replicase, triple gene block and coat protein genes [40]. (**C**) Bacterial system for antigen introduction in plant VLP structure [28]. Antigen (A) and CMV VLPs are obtained from separate bacterial cultures, purified, derivatized and joined in chemical coupling reaction. CMV *CP* gene contains T-cell epitope coding sequence (red box).

Several plant virus-based vaccines have been tested for tolerability, safety and efficacy in human clinical trials. The first such vaccine is an edible vaccine against rabies containing a recombinant antigen derived from rabies virus proteins (a fusion of peptides derived from the G and N proteins) incorporated in the N-terminus of the Alfalfa mosaic virus (AlMV). Introduction of the chimeric gene into the TMV-based plant vector and transient expression in spinach leaves resulted in AlMV-like particles exposing rabies epitopes. The inclusion of raw spinach leaves containing these VLPs in the diet of human volunteers led to significant antibody responses to rabies virus and plant virus carriers [48,49]. This pioneering study clearly demonstrates the potential of plant virus-based carriers for the generation of human vaccines. Later, several vaccine candidates were constructed based on AlMV carriers (Figure 3A [41,50]).

Malaria is one of the biggest health threats, causing hundreds of millions of cases and 435,000 deaths in 87 countries, especially in Africa [52]. The existing vaccine, RTS,S, has demonstrated only limited efficacy in clinical trials [17]. A recent publication [53] summarizes the results of a Phase I study assessing a malaria transmission-blocking vaccine based on a recombinant fusion of the *P. falciparum* antigen Pfs25 with the plant virus AlMV. Vaccine candidates produced in whole plants under cGMP conditions demonstrate acceptable safety and tolerability and induce antibodies against Pfs25, depending on the injected vaccine dose. However, the generated antibodies did not produce the expected reduction in *P. falciparum* transmission from mosquitoes to human host cells. The authors concluded that it is necessary to improve the formulation of the vaccine and increase the proportion of the Pfs25 antigen incorporated in plant VLPs.

Recently, other plant virus-derived VLPs have been suggested as active components for vaccines against malaria. The authors recommend the usage of thrombospondin-related adhesive protein (TRAP) as an antigen, which is necessary for *Plasmodium vivax* sporozoite motility and liver cell invasion. The vaccine was designed based on Cucumber mosaic virus (CMV) VLPs with chemically coupled TRAP antigen that were formulated with microcrystalline tyrosine (MCT) as an adjuvant. Vaccine formulations containing VLPs increased antibody production against TRAP compared to that produced by the antigen alone. The VLP-based vaccine formulated with MCT conferred significant protection in the challenge test with recombinant *P. berghei*, suggesting that MCT can be used as an advantageous adjuvant alternative for prophylactic VLP vaccines [45].

The next most important viral infection is influenza, which causes 250,000 to 500,000 deaths globally every year. Today, there are 26 licensed inactivated vaccines, some of which are produced routinely. However, existing vaccines do not ensure complete protection against the circulating influenza strains [54]. The reasons for low vaccine efficacy are the low levels of hemagglutination-inhibiting antibodies and influenza strain variations in the hemagglutinin (HA) sequence. For the improvement of flu vaccines, the use of stronger adjuvants and increased amounts of HA as well as the addition of new antigens (neuraminidase and matrix protein M2e) to influenza vaccines should increase efficacy [39].

Different peptide antigens, including peptides derived from nucleoproteins and M2e proteins, have been tested using plant viruses as vaccine carriers and adjuvants. Some of them demonstrated significant protection against challenges in different animal models (see examples in Supplementary Table S2). One experimental vaccine is currently being tested in a human trial that aims to evaluate the safety and reactogenicity of Papaya mosaic virus (PapMV) VLPs as adjuvants for use in a seasonal flu trivalent vaccine in 48 healthy volunteers [55]. The efficacy of the plant VLP-adjuvanted flu vaccine has not yet been reported. Earlier publications suggest that filamentous PapMV VLPs containing a consensus peptide of the influenza matrix M2e protein increase the survival of immunized mice if used as an adjuvant together with a trivalent inactivated flu vaccine. These results demonstrate the strong adjuvanting properties of filamentous plant VLPs for influenza vaccines [56].

Plant virus-based peptide vaccines are also being evaluated in veterinary trials. A vaccine based on Cowpea mosaic virus (CPMV) protects vaccinated dogs against lethal challenge with canine parvovirus [57]. Another vaccine against foot and mouth disease virus (FMDV) was designed using a bamboo mosaic virus (BaMV) vector and 37 AAs from the FMDV VP1 protein; all immunized swine were protected against FMDV challenge [58]. Another veterinary trial demonstrated vaccine safety and the protection of pigs against porcine circovirus (PCV) after vaccination with an inactivated CMV-based vaccine containing an incorporated PCV CP epitope [59].

## 3. Plant VLP-Derived Anticancer Vaccines

Cancer is one of the leading causes of death globally and was responsible for an estimated 9.6 million deaths in 2018 [60]. Currently, alongside surgery, chemotherapy and radiation, cancer immunotherapy has become an important component of cancer treatment.

Cancer immunotherapy has a more than 150-year history. It began with the first observations of the significant regression of sarcomas in patients after accidental infection by a *Streptococcus* bacterium. In 1891, William Coley, an American surgeon, used heat-inactivated bacteria to treat a large number of patients suffering from inoperable cancers. His therapy method resulted in the cure of more than 1000 patients. However, the method was later replaced with radiation and chemotherapy due to dangerous infection risks and the absence of reproducible results. The next important milestone in the development of cancer immunotherapy was the finding of Old et al. in 1959, which demonstrated the antitumor activity of the tuberculosis vaccine (BCG) in a mouse model. The vaccine has been introduced for the treatment of bladder cancer and has been used in clinics since the 1970s. These and other key events in cancer immunology are discussed in several recent review articles [61,62].

Several cell- and protein-based anticancer vaccines are approved for use in clinics or are being tested in late-stage clinical trials, such as autologous dendritic cell vaccines, recombinant virus-based vaccines, peptide-based vaccines, DNA vaccines, and human tumor whole-cell vaccines. However, for the most part, clinically tested anticancer vaccines have demonstrated limited or no efficacy in comparison to that of traditional treatments, requiring the development of new strategies and combinations of different approaches [63,64]. 

One such new approach for vaccination against cancer is based on the usage of nanoparticles, such as liposomes, carbon nanotubes, synthetic biodegradable and biocompatible polymers, inorganic nanoparticles, VLPs and different combinations of such particles [65].

VLPs represent a powerful and flexible tool for generation of the active components of cancer vaccines, as demonstrated in numerous preclinical studies (for review see [66]). We have already discussed the advantages of VLPs as vaccines, such as the multiplicity of antigens, the sizes of VLPs, which allow them to enter the lymphatic system, and their capability to encapsulate nucleic acids stimulating Toll-like receptors. As a result, VLPs are able to induce strong T cell responses, which is the most important requirement for a therapeutic vaccine against cancer. Moreover, VLP technology is already used for the prevention of cancer with Papilloma virus-derived VLPs, which efficiently protect immunized individuals against cervical cancer [66].

Plant viruses, including both native and recombinant viruses as well as their noninfectious derivatives (VLPs), have been considered as nanoparticle structures with antitumor activity since 2006, when McCormick et al. introduced a melanoma-specific peptide into TMV using recombinant fusion and chemical coupling [67,68] and tested both vaccines in tumor challenge models. The authors observed direct TMV uptake by dendritic cells and enhanced production of interferon ɣ (IFNg). Interestingly, the vaccine prepared by the chemical coupling of peptides to TMV ensured better survival of the animals than the recombinant vaccine when both vaccine variants were formulated with the CpG DNA adjuvant. 

The importance of vaccine formulation was also demonstrated in a recent study using icosahedral CMV VLPs with chemically coupled p33 peptide derived from lymphocytic choriomeningitis virus (LCMV). The vaccine was formulated with MCT, CpG and alum adjuvants and tested in an aggressive mouse melanoma model.

VLPs adjuvanted with MCT effectively retarded the development of the tumor. The effect was comparable when CpG DNA was used as the adjuvant, whereas alum was ineffective in slowing down tumor growth [69].

A recent study suggests a highly interesting immunotherapy approach. Three different icosahedral plant virus-derived nanoparticles with chemically coupled breast cancer epitopes efficiently elicited the formation of antibodies against the HER2 receptor, which is overexpressed in breast cancer cells. Additionally, all three vaccines stimulated T-cell-mediated immune responses when tested separately. Sequential use of these vaccines reduced the immune responses against VLP carriers and improved the formation of antibodies against the HER2 peptide. This suggested prime-boost strategy considerably reduced tumor development and enhanced the survival rate in a mouse tumor model. The study clearly demonstrated that the Th1-type immune response, including the formation of IgG2a antibodies, the secretion of IFNg and the activation of CD4+/CD8+ T-cells, is the most important factor for immunotherapy. Interestingly, plant VLPs derived from CPMV demonstrated better immunotherapeutic properties than those derived from Cowpea chlorotic mottle virus (CCMV) and Sesbania mosaic virus (SeMV). Therefore, additional studies are needed to understand the differences between different plant VLPs used as carriers for anticancer vaccines [70]. Other examples of plant virus-based cancer vaccines with the corresponding literature citations are summarized in Supplementary Table S3.

The analysis of several recent publications reveals the potential application of unmodified plant VLPs without any introduced antigens as cancer immunotherapy agents [71,72]. In one study, the authors compared differently prepared nanoparticles derived from CPMV as immunotherapeutic agents in a murine ovarian cancer model. Native CPMV particles containing viral RNA induced a more pronounced therapeutic effect and the survival of experimental animals in comparison to empty CPMV VLPs produced in plant or insect cells. The enhanced immunomodulatory effect apparently is due to the presence of encapsidated ssRNA in native CPMV virions, which activates Toll-like receptors 7/8. Interestingly, the chosen recombinant host can also influence the immune-stimulating properties of produced plant VLPs [72].

In another study, structural variants of plant virus-derived nanoparticles from several species were used as in situ vaccines, such as icosahedral CPMV and three variants of TMV (native, in vitro RNA-templated assembly of short TMV, and spherical TMV produced after thermal treatment of native virions). The results suggest the superior immune-stimulating properties of CPMV compared with those of other structural variants of TMV and confirm that antigen multiplicity is one of the most important factors involved in eliciting a strong immune response. One possible explanation for the enhanced immune-stimulating properties of CPMV in a mouse melanoma model is the ability of VLPs to recruit monocytes into the tumor microenvironment, leading to the infiltration of neutrophils and natural killer cells and resulting in tumor growth inhibition. Authors suggest that the intrinsic properties of some plant viruses allow them to be developed as cancer vaccines for clinical use; however, the detailed mechanisms of immune stimulation remain to be elucidated [71].

## 4. Plant VLP-Derived Vaccines against Allergies, Autoimmune Diseases and Other Diseases

Vaccinations are also highly promising for the treatment of allergies and autoimmune and neurodegenerative diseases, as demonstrated in the latest studies. Similar to antimicrobial and anticancer vaccines, plant viruses and VLPs can serve as carrier structures for corresponding antigens, which are important in disease development.

One of the most challenging diseases in terms of therapy development is Alzheimer’s disease (AD). More than 40 million people worldwide are suffering from dementia, which is caused by the formation of plaques containing proteolytic fragments of amyloid precursor protein Aβ (AA 1-42). Existing therapies and experimental approaches, including the use of monoclonal antibodies and vaccines, have only a small impact on disease progression. According to a recent opinion, vaccination should be prophylactic; the levels of specific antibodies have to be sufficiently high for the targeting of oligomeric species of Aβ peptides, and the sizes of the epitopes included in vaccines have to be less than 8 AA to prevent the stimulation of pathogenic T-cell responses [73]. 

The concept of AD vaccines has existed for more than 15 years; however, the first clinical trial using a vaccine containing aggregated Aβ peptide (AA 1-42) resulted in cases of T-cell-mediated brain inflammation [74]. Later, an experimental vaccine consisting of Aβ peptide and a bacteriophage Qβ conjugate produced strong antibody responses without significant T-cell responses in mice [75].

Plant viruses and derived VLPs are also considered as epitope carriers for Alzheimer vaccines. Potential vaccine candidates have been generated using infectious CMV fused with Aβ peptides purified from plant biomass [76]. Recently, we constructed a plant VLP platform (Figure 3C) based on the same CMV by the genetic incorporation of a universal T-cell epitope in the interior of particles and the chemical coupling of Aβ1-6 peptide to the VLP surface. Sera obtained from immunized mice were shown to recognize Alzheimer plaques in human brain sections, suggesting that the CMV-Aβ(1-6) vaccine induced the production of specific antibodies [28].

Allergic disorders have been among of the most common chronic diseases in Europe in recent decades [77]. Patients suffering from allergies have to avoid allergens and use anti-histamine medications. Alternatively, allergen-specific immunotherapy (AIT) is the only available treatment for the reduction of allergy symptoms. For therapy, crude allergen extracts containing a mixture of native allergenic proteins are used. The typical disadvantages of AIT are the risk of anaphylactic reactions, the long duration of therapy, the low quality of the natural extracts used in AIT and the unsatisfactory efficacy of the treatment. 

Peanut allergy is the most frequent cause of anaphylactic reactions and death among food allergies. There is currently no safe and effective therapy for peanut allergy, especially for patients with a severe allergy. Recently, we constructed several plant virus-based, immunologically optimized vaccines for peanut allergy by chemical coupling of peanut allergens Ara h 1 and Ara h 2 as well as mixture of proteins purified from roasted peanut extract to CMV VLPs. The resulting vaccines did not cause allergic reactions and induced specific IgG antibodies to protect peanut-sensitized mice against anaphylactic shock. Notably, immunizations with single allergen-containing VLPs ensured protection against challenge with the complex allergen mixture, suggesting a new vaccination strategy for the treatment of peanut allergy [78]. Other examples of VLP usage in the treatment of allergic diseases are summarized in a recently published review article [79]. 

Allergies to cats affect more than 10% of the human population, and the prevalence is increasing [80]. Similar to that of food allergies, current cat allergy treatment includes the avoidance of exposure, the use of anti-histamines and steroids and long-term subcutaneous immunotherapy. All these measures are only partially effective in eliminating allergic reactions; additionally, immunotherapy is bound to result in safety issues. The predominant cat allergen is the secretoglobulin Fel d 1, which is secreted by cat sebaceous and salivary glands. We generated a CMV-based vaccine containing recombinant Fel d 1 allergen using chemical coupling and demonstrated that VLP coupling effectively reduces allergic reactions, stimulates the formation of Fel d 1-specific IgGs and protects sensitized mice against anaphylactic shock [76]. Moreover, Bachmann and collaborators proposed a new strategy for the treatment of Fel d 1 allergy involving immunizing cats against their own Fel d 1 allergen. The induced anti–Fel d 1 antibodies exhibited a strong neutralization ability and might result in reduced symptoms in allergic cat owners [81].

Monoclonal antibodies (mAbs) produced by immortalized hybridoma cells represent a new way to treat different diseases. Since the approval of the first mAb preventing kidney transplant rejection in 1992, numerous mAbs have been used in clinics for treatment of cancers, bacterial and viral infections, and various cardiovascular, respiratory, neurological and autoimmune diseases [82,83]. MAbs have been proven to be efficient agents for treating chronic inflammatory diseases via the selective inhibition of cytokines, which are excessively produced in several disease conditions. Taking into account the high costs and side effects of mAb therapies, there is significant interest in replacing mAb treatments with active immunization against autologous proteins, including interleukins [84,85]. 

Based on this idea, we generated an IL17-containing plant VLP-based vaccine and tested it in a mice psoriasis model [28]. The vaccinated mice demonstrated a similar reduction in psoriatic symptoms compared to that in animals passively immunized with IL17A antibody. Moreover, the vaccination elicited protective effects in suboptimal conditions, such as those involving older mice or low vaccine doses. 

The knowledge obtained from successful studies of human vaccinations and mAb treatments can be transferred to the development of veterinary vaccines. It is well known that, for example, allergic hypersensitivity is linked to the activation of eosinophils and the enhanced production of interleukin 5 (IL5) by Th2 cells. The eosinophil count can be effectively reduced by a specific humanized anti-IL5 mAb, which is used for treatment of human asthma [86]. Active vaccination with a plant VLP-IL5 conjugate instead of a mAb induced a potent IL5 antibody response in horses, reducing the symptoms of insect bite hypersensitivity. Simultaneously, vaccination did not significantly influence the blood eosinophil count and the parasitic load in vaccinated horses [87,88].

Another example of a CMV-based therapeutic vaccine is a vaccine reducing the production of IL-31 in dogs and horses suffering from itching during atopic dermatitis or insect bite hypersensitivity. In both animals, the vaccine is well tolerated and improves the disease symptoms [89,90]. 

In the last few years, nerve growth factor (NGF), a key molecule involved in the regulation of neuronal regeneration during injury and pain perception, has been suggested as a promising target for osteoarthritis (OA) treatment. In humans, monoclonal antibodies against NGF significantly suppress pain associated with late-stage OA. Based on this, we constructed a CMV-based NGF vaccine and demonstrated its therapeutic efficacy by showing that it alleviates spontaneous pain behavior in surgically induced murine OA [91].

## 5. Conclusions

Vaccine production is one of the most challenging industrial processes. Several important factors influence vaccine development and marketing. One of them is the time required to bring a new vaccine from the development phase to the market, which can exceed 15 years, including 7 years on average for the design, construction, validation and commencement of industrial manufacture. The next factor is the significant human and financial resources necessary for the development and sustainable on-demand production of vaccines. The vaccine industry has to continuously solve problems, such as long life cycles of vaccines, high facility costs and the complexity of global vaccine demand [92,93]. Long vaccine development periods are an additional challenge negatively influencing the availability of vaccines against emerging infectious diseases, such as Ebola virus, SARS-CoV, Zika virus, Chickungunja virus and others [94]. Moreover, the chosen antigen structure used for vaccine manufacturing can be different from the corresponding native antigen, reducing or even preventing the neutralizing ability of the generated antibodies. This aspect critically influences the development process of vaccines against pathogens, especially against infectious agents with high genetic variation. HIV is an example of such a pathogen, against which there is no effective vaccine despite many years of effort by academic scientists and the vaccine industry [95]. One other challenging aspect that should be mentioned here as a factor that is possibly important for vaccine development in the future is the fact that the latest studies suggest that the microbiome in the digestive track of recipients can influence the efficacy of vaccines. The effect is observed both in humans and in laboratory mice, suggesting the need for further investigation [96].

As shown in this review, plant viruses and their noninfectious derivatives (VLPs) have been intensively studied as immunologically active multivalent structures useful for the generation of new prophylactic and therapeutic vaccines against human or animal infectious agents, cancers and autoimmune diseases. When compared with other VLPs, plant VLP-based vaccines have additional advantages, such as flexibility in vaccine construction, the ease of VLP production and purification, stability and the low risk of preexisting immunity. All these properties make plant viruses an attractive alternative to animal and human VLPs.

Today, no plant virus-based vaccines are commercially available. However, several vaccine platforms have been developed that allow the construction of new vaccines in a comparably short period of time. Some experimental vaccine platforms tolerate the incorporation of large antigens and even full-sized proteins in viral structures without influencing the particle morphology and immune-stimulating properties. As the native spatial structures of recombinant antigens are highly important for eliciting antibodies with neutralizing activity and long-lasting immunity [97,98], future recombinant VLP vaccines must contain multiple copies of correctly folded antigens as well as different immunostimulating components, including T-cell epitopes and nucleic acids.

The achievements discussed here suggest that vaccines based on plant virus-based carriers are very useful for addressing different challenges in vaccine construction and will be developed into new and approved prophylactic and therapeutic vaccines for human and veterinary use in the coming years.

## Supplementary Materials

The following are available online at https://www.mdpi.com/1999-4915/12/3/270/s1.
