# Supplementary material for: Recent Advances in the Use of Plant Virus-Like Particles as Vaccines
Source: Viruses. 2020 Feb 28;12(3):270. doi: 10.3390/v12030270 (PMC7150928; doi:10.3390/v12030270)
Supplement: Supplementary file 1 [file viruses-12-00270-s001.zip › Suppl_table_1_Vaccines.docx]

Supplement Table 1. List of diseases and available vaccines according to data from the World Health Organization (https://www.who.int/immunization/diseases/en/).

**Disease Vaccine antigen Method Valence Administration**

[Cholera](https://www.who.int/immunization/diseases/cholera/en/) *Vibrio cholerae;* whole-cell thermally or chemically inactivated monovalent oral

cells

[Dengue](https://www.who.int/immunization/diseases/dengue/en/) Dengue virus; PrM and E proteins live attenuated, recombinant tetravalent injections

[Diphtheria](https://www.who.int/immunization/diseases/diphtheria/en/) *Corynebacterium diphtheria;* toxoid inactivated toxin monovalent, in combination injections

with tetanus toxoid

[Hepatitis A](https://www.who.int/immunization/diseases/hepatitisA/en/) Hepatitis A virus; whole virus chemically inactivated monovalent injections

[Hepatitis B](https://www.who.int/immunization/diseases/hepatitisB/en/) Hepatitis B virus; surface antigen recombinant VLPs monovalent injections

[Hepatitis E](https://www.who.int/immunization/diseases/hepatitisE/en/) Hepatitis E; structural protein recombinant subunits (VLPs) monovalent injections

*Haemophilus influenzae H. influenza* B; polysaccharides protein carrier conjugates monovalent injections

[Human papillomavirus](https://www.who.int/immunization/diseases/hpv/en/)  HPV; L1 protein recombinant subunits (VLPs) bi-, tetra and nonavalent injections

[Influenza](https://www.who.int/immunization/diseases/influenza/en/) whole virus inactivated or live attenuated tri- and quadrivalent injections, nasal sprays

[Japanese encephalitis](https://www.who.int/immunization/diseases/japanese_encephalitis/en/) JEV genotype III; whole virus inactivated or live attenuated, monovalent injections

recombinant

[Malaria](https://www.who.int/immunization/research/development/malaria/en/) *P. falciparum*; CSP protein recombinant CSP fusion with monovalent injections

HBsAg subunits (VLPs)

[Measles](https://www.who.int/immunization/diseases/measles/en/) Measles virus; whole virus live attenuated monovalent or in injections

combinations with others

[Meningococcal meningitis](https://www.who.int/immunization/diseases/meningitis/en/) *N. meningitidis;*  polysaccharides protein carrier conjugates bi-, tri- or quadrivelent injections

**Disease Vaccine antigen Method Valence Administration**

[Mumps](https://www.who.int/immunization/diseases/mumps/en/) Mumps virus; whole virus live attenuated monovalent or in injections

combinations with others

[Pertussis](https://www.who.int/immunization/diseases/pertussis/en/) *Bordetella pertussis;* whole cell or chemically inactivated cells in combinations with others injections

individual antigens (toxin, fimbriae, etc.) or purified subunits

[Pneumococcal disease](https://www.who.int/immunization/diseases/pneumococcal/en/) *Streptococcus pneumoniae;* polysaccharides protein carrier conjugates heptavalent, decavalent or injections

tridecavalent

[Poliomyelitis](https://www.who.int/immunization/diseases/poliomyelitis/en/) Poliovirus; whole virus live attenuated or inactivated mono-, bi- or trivalent injections

or oral

[Rabies](https://www.who.int/immunization/diseases/rabies/en/) Rabies lyssavirus; whole virus live attenuated and/or inactivated monovalent injections

or oral

[Rotavirus](https://www.who.int/immunization/diseases/rotavirus/en/) Rotavirus; whole virus live (reassotment) or live attenuated monovalent or pentavalent oral

[Rubella](https://www.who.int/immunization/diseases/rubella/en/) Rubella virus; whole virus live attenuated monovalent or in combinations injections

with others

[Tetanus](https://www.who.int/immunization/diseases/tetanus/en/) *Clostridium tetani;* toxoid chemically inactivated toxoid in combinations with others injections

[Tick-borne encephalitis](https://www.who.int/immunization/diseases/tick_encephalitis/en/) TBEV virus; whole virus chemically inactivated monovalent injections

[Tuberculosis](https://www.who.int/immunization/diseases/tuberculosis/en/) *Mycobacterium tuberculosis;* whole cells live attenuated monovalent intradermal

[Typhoid](https://www.who.int/immunization/diseases/typhoid/en/) *Salmonella typhi;* whole cells poysaccharides or protein conjugates monovalent injections

or polysaccharides or live attenuated cells or oral

[Varicella](https://www.who.int/immunization/diseases/varicella/en/) Varicella-zoster virus; whole virus live attenuated monovalent or in injections

combinations with others

[Yellow Fever](https://www.who.int/immunization/diseases/yellow_fever/en/) Yellow fever virus; whole virus live attenuated monovalent injections
