# Supplementary material for: Recent Advances in the Use of Plant Virus-Like Particles as Vaccines
Source: Viruses. 2020 Feb 28;12(3):270. doi: 10.3390/v12030270 (PMC7150928; doi:10.3390/v12030270)
Supplement: Supplementary file 1 [file viruses-12-00270-s001.zip › Suppl_Table_2_vaccines_infect_diseases.docx]

Supplement Table 2. Examples of vaccine candidates against infectious agents based on plant virus carriers (updated from Balke I., Zeltins A. (2019) Adv.Drug Deliv. Rev. 145:119-129)

| **Vaccine candidate** | **Antigen** | **Plant virus / VLP** | **Expression system** | **Immunological data** | **Reference** |
| --- | --- | --- | --- | --- | --- |

Anthrax *Bacillus anthracis* protective antigenic peptides CPMV Plant Partial toxin neutralization in mice [1]

Canine N-terminal fragment of L2 protein (AA 61-171) TMV Plant Streptavidin-L2 fragment bound to TMV [2]

papillomavirus enhances the immunogenicity in mice

Canine CPV VP2 protective epitope (15 AA) PPV Plant CPV neutralizing antibodies in mice [3,4]

parvovirus and rabbits

CPV VP2 protective epitope (AA 3-19) CPMV Plant CPV protective antibodies in mice, dogs [5-7]

protected after subcutaneous or intranasal

vaccination

CPV VP2 protective epitope (15 AA) PRSV  *E. coli* Strong immune response against epitope [8]

in mice

Different neutralizing and T-cell epitopes PhMV *E. coli*  Protective antibodies against CPV [9]

of CPV in guinea pigs and dogs

Classical swine CSFV E2 peptides (40 and 70 AA) PVX Plant Positive Western blots using antibodies [10]

fever virus from immunized rabbits

Cottontail rabbit CRPV L2 (AA 94-122) TMV Plant Strong protection against experimental [11]

papillomavirus papillomavirus infection

| **Vaccine candidate** | **Antigen** | **Plant virus / VLP** | **Expression system** | **Immunological data** | **Reference** |
| --- | --- | --- | --- | --- | --- |

Dengue virus EB4 protein domain (45 AA) CGMMV Plant data not found [12]

type 2

Foot and mouth FMDV VP1 epitope (25 AA) CPMV Plant Epitope-containing VLPs react with specific [13-15]

disease virus sera

FMDV VP1 peptides (AA 144-152 and 142-150); TMV Plant Vaccination protects guinea pigs and swine [16,17]

fused epitope (AA 200-213 and 142-150) against FMDV infection

Different FMDV epitopes PhMV *E. coli* Epitope-containing VLPs react with sera [18]

from infected animals

FMDV VP1 epitope (AA 128-164) BaMV Plant Vaccination protects against FMDV [7]

challenge

Different FMDV VP1 peptides TNV-A Plant Strong immune response against VP1 [19]

epitopes in mice

*Francisella* OmpA (47 kDa), DnaK (70 kDa), Tul4 (17 kDa) TMV Plant Multiconjugate vaccine partially protects [20]

*tularensisis* mice against lethal doses of *F.tularensis*

Tetra-antigen vaccine, CpG as adjuvant TMV Plant (TMV) Two vaccine doses protect 80% of mice [21]

*E.coli* (antigens) from lethal pathogen challenge

Hepatitis B HBsAg (AA 111-141) CGMMV Plant VLPs stimulate *in vitro* cultured human [22]

virus PBMC from pre-immunized donors

| **Vaccine candidate** | **Antigen** | **Plant virus / VLP** | **Expression system** | **Immunological data** | **Reference** |
| --- | --- | --- | --- | --- | --- |

HBV preS1 epitope (AA 20-41) PVY *E. coli* Strong immune response against [23]

preS1 epitope in mice

Hepatitis C HCV neutralizing “mimotope” R9 (27 AA) CMV Plant VLPs react with immune sera from [24-28]

virus HCV-positive patients (>80%)

HCV E2 epitope (AA 511-530) PapMV *E. coli* Humoral response in mice depends on [29]

antigen multimerization;

VLPs react with immune sera from

HCV-positive patients

HCV neutralizing “mimotope” R9 (27 AA) PVX Plant VLPs react with immune sera from [30]

HCV-positive patients (35%)

Human immuno- HIV-1 gp41 epitope (AA 731-752) CPMV Plant VLPs stimulate the formation of [31-34];

deficiency virus neutralizing antibodies in mice

HIV-1 gp120 V3 loop (13 AA) TBSV Plant VLPs react with immune sera from [35]

HIV-1-positive patients

HIV-1 gp41 epitope (6 AA) PVX Plant sera from VLP-immunized mice [36]

demonstrate HIV-1-neutralizing activity

HIV-1 gp41 epitopes CdMV *E. coli* VLPs react with immune sera from [37]

HIV-1-positive patients

HIV-1 gp41 epitope (6 AA) AMCV Plant data not found [38]

| **Vaccine candidate** | **Antigen** | **Plant virus / VLP** | **Expression system** | **Immunological data** | **Reference** |
| --- | --- | --- | --- | --- | --- |

Human HPV16 E7 peptide (AA 44-60) PVA Mammalian DNA vaccination protect mice [39]

papilloma virus (293T cells) against tumor development,

HPV E7ggg oncoprotein, PVX  *E. coli,* plant Strong immune response against L2 [40-43];

L2 peptide (AA 108-120) epitope in mice

Human HRV-14 VP1 epitope (AA 85-98) CPMV Plant VLPs are immunogenic in rabbits [44,45]

rhinovirus

Infectious IBDV VP2 epitope (AA 207–224) BaMV Plant VLPs protect immunized chickens from [46]

bursal disease virus challenge

virus

IBDV neutralizing epitopes PhMV *E.coli* VLPs recognized by IBDV antibodies [47]

Influenza Influenza M1 epitope (AA 57-65); PapMV *E. coli* M2e-VLPs adjuvanted with unmodified [48-54];

viruses Influenza A (H1N1) VLPs lead to 100% protection against;

M2e peptides (AA 2-24 / 6-14); lethal challenge in mice; stable

HA11 epitope (9 AA); NP epitope (AA 147-155) VLPs are more immunogenic;

M2e-VLPs serve as efficacious adjuvant for

trivalent inactivated flu vaccine

Nucleoprotein (NP), M2e peptide PapMV *E.coli* Combination of the NP nanoparticles [55]

with the PapMV-M2e nanoparticles

protects mice from infectious challenges

by influenza strains H1N1 and H3N2

| **Vaccine candidate** | **Antigen** | **Plant virus / VLP** | **Expression system** | **Immunological data** | **Reference** |
| --- | --- | --- | --- | --- | --- |

Influenza Nucleoprotein (NP) PapMV *E.coli;* NP coupled to PapMV VLPs [56]

Viruses Sortase A improved significantly the humoral

mediated and CTL immune response

coupling

Influenza A NP peptide (AA 336-374) PVX Plant VLPs activate specific CD8+ cells [57]

Influenza M2e peptides AltMV Plant data not found [58]

Influenza M2e peptide (24 AA) CCMV *E. coli;* data not found [59,60]

*P. fluorescens*

Influenza M2e peptide MaMV *E. coli* OmpC porine-adjuvanted VLPs increase [61]

the immune response in mice and dogs

Influenza HA protein (ca. 60 kDa) TMV Plant Squalene-adjuvanted HA-VLPs lead to [62]

100% protection against virus challenge

in mice

Influenza AH1N1 HA peptide (AA 87-120) PRSV *E. coli* VLPs demonstrate strong adjuvant [63]

properties in immunized mice

*Yersinia pe*s*tis* Virulence factors F1 (17.6 kDa) TMV Plant Chemically conjugated LcrV and F1 [64]

and LcrV (37 kDa) proteins to viral particles protect

mice against lethal challenge

Japanese JEV envelope protein PhMV *E. coli* Refolded VLPs generate neutralizing [65]

encephalitis epitopes (42 AA) antibodies in mice

virus

JEV EDIII domain (ca 12 kDa) BaMV Plant EDIII-VLPs generate neutralizing antibodies [66]

in mice

| **Vaccine candidate** | **Antigen** | **Plant virus / VLP** | **Expression system** | **Immunological data** | **Reference** |
| --- | --- | --- | --- | --- | --- |

Lymphocytic LCMV p33 CTL epitope (9 AA) PapMV *E. coli* p33-VLPs induce protective cell-mediated [67]

choriomeningitis antiviral immunity

virus

Malaria *P.vivax* and *P.yoelii* peptides from TMV Plant Specific mAb’s react with peptide [68]

imunodominant protein containing TMV

*P. falciparum* Pfs25 (25 kDa) AlMV Plant Pfs25-VLPs block *P.falciparum* transmission [69]

in mice

*P. falciparum* Pfs25 (25 kDa) AlMV Plant Produced under cGMP Pfs25-VLPs induced [70]

10% of antigen Pfs25-specific IgG in a dose dependent

incorporation manner; 44 human participants in Phase 1

study; low transmission reducing activity

*P. vivax* TRAP protein (AA 25-493) CMV *E. coli* (VLPs) Chemically conjugated TRAP to VLPs [71]

Mammalian protects mice against *P. berghei* challenge

(TRAP)

Mink enteritis MEV VP2 epitope (AA 3-19) CPMV Plant VLPs protect minks against virus challenge [72]

virus

Murine hepatitis MHV 5B19 epitope (AA 899-913) TMV Plant 5B19-VLPs protect mice against lethal [73]

virus virus challenge

Newcastle NDV F epitope (AA 65-81) and CMV Plant Polyclonal antibodies obtained after [74-76]

disease virus HN (AA 346-353) chicken immunization with HN-VLPs

recognize native NDV

| **Vaccine candidate** | **Antigen** | **Plant virus / VLP** | **Expression system** | **Immunological data** | **Reference** |
| --- | --- | --- | --- | --- | --- |

Poliovirus PV type 3 VP1 epitope (8 AA) TMV *E. coli* Epitope-containing VLPs induce the virus- [77]

neutralizing antibodies in rats

PV type 1 peptide (15 AA) derived TMV Plant VLPs induce antibodies against [78]

from VP1 and VP3 from peptide in immunized mice

Porcine PCV type 2 CP epitope (AA 19-28) PRSV *E. coli* Strong immune response against peptide [79]

circovirus in mice

PCV type 2 CP epitope (AA 224-233) CMV Plant Immunized pigs partially protected [80]

against PCV challenge

Porcine neutralizing epitope of PRRSV glycoprotein 5 CGMMV Plant no data found [81]

reproductive 10% of antigen

and respiratory incorporated

syndrome virus

*Pseudomonas* OM-F protein tandem peptide (34 AA) CPMV Plant Peptide specific mice antibodies [82,83]

*aeruginosa* recognize all immunotypes of

*P.aeruginosa;* immunized mice are

partially protected against challenge

OM-F protein peptides TMV Plant Peptide specific mice antibodies [84,85]

recognize all immunotypes of

*P.aeruginosa;* immunized mice are

protected against challenge

| **Vaccine candidate** | **Antigen** | **Plant virus / VLP** | **Expression system** | **Immunological data** | **Reference** |
| --- | --- | --- | --- | --- | --- |

Rabies Antigenic peptide Drg24 / T-cell epitope 31D AlMV Plant Peptide-containing VLPs elicit [86,87]

(AA 253-275 / AA 404-418) virus-neutralizing antibodies; edible

vaccine serves as oral booster in

human volunteers

Adjuvant for rabies vaccine TMV Plant VLPs enhance the protection of mice [88]

spherical VLPs against rabies

Respiratory RSV RhoA protein peptide (AA 80-94) AlMV Plant data not found [89]

syncytial virus

Ricin toxin Ricine toxin chain A peptide (16 AA) TBSV Insect Antibodies from immunized mice recognize [90]

native toxin

Rubella virus RV E1 tetrapetide (32 AA x 4) TMV Plant Strong humoral immune response in mice [91]

against rubella

*Staphylococcus* FnBP D2 peptide (AA 1-38) PVX Plant Sera from peptide-VLP immunized mice [92]

*aureus* inhibit the binding of fibronectin to

immobilized FnBP

FnBP D2 peptide (AA 1-30) CPMV Plant Sera from peptide-VLP immunized rats [92]

inhibit the binding of *S. aureus*

to immobilized FnBP

*Streptococcus* S9 peptide mimicking capsular CCMV Yeast *P. pastoris* Peptide conjugated VLPs elicits [93]

group B polysaccharide (13 AA) Th1 response

S9 peptide mimicking capsular CPMV Plant Peptide conjugated VLPs elicits [93]

polysaccharide (13 AA) Th1 response

| **Vaccine candidate** | **Antigen** | **Plant virus / VLP** | **Expression system** | **Immunological data** | **Reference** |
| --- | --- | --- | --- | --- | --- |

Zika virus ZIKV envelope protein CMV *E.coli* Vaccine induces high levels of [94]

domain III chemical specific IgGs able to neutralize

coupling ZIKV *in vitro*

**References**

1. McComb, R.C.; Ho, C.L.; Bradley, K.A.; Grill, L.K.; Martchenko, M. Presentation of peptides from Bacillus anthracis protective antigen on Tobacco Mosaic Virus as an epitope targeted anthrax vaccine. *Vaccine* **2015**, *33*, 6745-6751, doi:10.1016/j.vaccine.2015.10.075.

2. Smith, M.L.; Lindbo, J.A.; Dillard-Telm, S.; Brosio, P.M.; Lasnik, A.B.; McCormick, A.A.; Nguyen, L.V.; Palmer, K.E. Modified tobacco mosaic virus particles as scaffolds for display of protein antigens for vaccine applications. *Virology* **2006**, *348*, 475-488, doi:10.1016/j.virol.2005.12.039.

3. Fernandez-Fernandez, M.R.; Martinez-Torrecuadrada, J.L.; Casal, J.I.; Garcia, J.A. Development of an antigen presentation system based on plum pox potyvirus. *FEBS Lett* **1998**, *427*, 229-235.

4. Fernandez-Fernandez, M.R.; Martinez-Torrecuadrada, J.L.; Roncal, F.; Dominguez, E.; Garcia, J.A. Identification of immunogenic hot spots within plum pox potyvirus capsid protein for efficient antigen presentation. *J Virol* **2002**, *76*, 12646-12653.

5. Nicholas, B.L.; Brennan, F.R.; Hamilton, W.D.; Wakelin, D. Effect of priming/booster immunisation protocols on immune response to canine parvovirus peptide induced by vaccination with a chimaeric plant virus construct. *Vaccine* **2003**, *21*, 2441-2447.

6. Deenick, E.K.; Hasbold, J.; Hodgkin, P.D. Decision criteria for resolving isotype switching conflicts by B cells. *Eur J Immunol* **2005**, *35*, 2949-2955, doi:10.1002/eji.200425719.

7. Yang, C.D.; Liao, J.T.; Lai, C.Y.; Jong, M.H.; Liang, C.M.; Lin, Y.L.; Lin, N.S.; Hsu, Y.H.; Liang, S.M. Induction of protective immunity in swine by recombinant bamboo mosaic virus expressing foot-and-mouth disease virus epitopes. *BMC Biotechnol* **2007**, *7*, 62, doi:10.1186/1472-6750-7-62.

8. Chatchen, S.; Juricek, M.; Rueda, P.; Kertbundit, S. Papaya ringspot virus coat protein gene for antigen presentation in Escherichia coli. *J Biochem Mol Biol* **2006**, *39*, 16-21.

9. Chandran, D.; Shahana, P.V.; Rani, G.S.; Sugumar, P.; Shankar, C.R.; Srinivasan, V.A. Display of neutralizing epitopes of Canine parvovirus and a T-cell epitope of the fusion protein of Canine distemper virus on chimeric tymovirus-like particles and its use as a vaccine candidate both against Canine parvo and Canine distemper. *Vaccine* **2009**, *28*, 132-139, doi:10.1016/j.vaccine.2009.09.093.

10. Marconi, G.; Albertini, E.; Barone, P.; De Marchis, F.; Lico, C.; Marusic, C.; Rutili, D.; Veronesi, F.; Porceddu, A. In planta production of two peptides of the Classical Swine Fever Virus (CSFV) E2 glycoprotein fused to the coat protein of potato virus X. *BMC Biotechnol* **2006**, *6*, 29, doi:10.1186/1472-6750-6-29.

11. Palmer, K.E.; Benko, A.; Doucette, S.A.; Cameron, T.I.; Foster, T.; Hanley, K.M.; McCormick, A.A.; McCulloch, M.; Pogue, G.P.; Smith, M.L., et al. Protection of rabbits against cutaneous papillomavirus infection using recombinant tobacco mosaic virus containing L2 capsid epitopes. *Vaccine* **2006**, *24*, 5516-5525, doi:10.1016/j.vaccine.2006.04.058.

12. Teoh, P.G.; Ooi, A.S.; AbuBakar, S.; Othman, R.Y. Virus-specific read-through codon preference affects infectivity of chimeric cucumber green mottle mosaic viruses displaying a dengue virus epitope. *J Biomed Biotechnol* **2009**, *2009*, 781712, doi:10.1155/2009/781712.

13. Usha, R.; Rohll, J.B.; Spall, V.E.; Shanks, M.; Maule, A.J.; Johnson, J.E.; Lomonossoff, G.P. Expression of an animal virus antigenic site on the surface of a plant virus particle. *Virology* **1993**, *197*, 366-374, doi:10.1006/viro.1993.1598.

14. Taylor, K.M.; Porta, C.; Lin, T.; Johnson, J.E.; Barker, P.J.; Lomonossoff, G.P. Position-dependent processing of peptides presented on the surface of cowpea mosaic virus. *Biol Chem* **1999**, *380*, 387-392, doi:10.1515/BC.1999.051.

15. Gopinath, K.; Wellink, J.; Porta, C.; Taylor, K.M.; Lomonossoff, G.P.; van Kammen, A. Engineering cowpea mosaic virus RNA-2 into a vector to express heterologous proteins in plants. *Virology* **2000**, *267*, 159-173, doi:10.1006/viro.1999.0126.

16. Wu, L.; Jiang, L.; Zhou, Z.; Fan, J.; Zhang, Q.; Zhu, H.; Han, Q.; Xu, Z. Expression of foot-and-mouth disease virus epitopes in tobacco by a tobacco mosaic virus-based vector. *Vaccine* **2003**, *21*, 4390-4398.

17. Jiang, L.; Li, Q.; Li, M.; Zhou, Z.; Wu, L.; Fan, J.; Zhang, Q.; Zhu, H.; Xu, Z. A modified TMV-based vector facilitates the expression of longer foreign epitopes in tobacco. *Vaccine* **2006**, *24*, 109-115, doi:10.1016/j.vaccine.2005.09.060.

18. Hema, M.; Nagendrakumar, S.B.; Yamini, R.; Chandran, D.; Rajendra, L.; Thiagarajan, D.; Parida, S.; Paton, D.J.; Srinivasan, V.A. Chimeric tymovirus-like particles displaying foot-and-mouth disease virus non-structural protein epitopes and its use for detection of FMDV-NSP antibodies. *Vaccine* **2007**, *25*, 4784-4794, doi:10.1016/j.vaccine.2007.04.023.

19. Zhang, Y.; Li, J.; Pu, H.; Jin, J.; Zhang, X.; Chen, M.; Wang, B.; Han, C.; Yu, J.; Li, D. Development of Tobacco necrosis virus A as a vector for efficient and stable expression of FMDV VP1 peptides. *Plant Biotechnol J* **2010**, *8*, 506-523, doi:10.1111/j.1467-7652.2010.00500.x.

20. Banik, S.; Mansour, A.A.; Suresh, R.V.; Wykoff-Clary, S.; Malik, M.; McCormick, A.A.; Bakshi, C.S. Development of a Multivalent Subunit Vaccine against Tularemia Using Tobacco Mosaic Virus (TMV) Based Delivery System. *PLoS One* **2015**, *10*, e0130858, doi:10.1371/journal.pone.0130858.

21. Mansour, A.A.; Banik, S.; Suresh, R.V.; Kaur, H.; Malik, M.; McCormick, A.A.; Bakshi, C.S. An Improved Tobacco Mosaic Virus (TMV)-Conjugated Multiantigen Subunit Vaccine Against Respiratory Tularemia. *Front Microbiol* **2018**, *9*, 1195, doi:10.3389/fmicb.2018.01195.

22. Ooi, A.; Tan, S.; Mohamed, R.; Rahman, N.A.; Othman, R.Y. The full-length clone of cucumber green mottle mosaic virus and its application as an expression system for Hepatitis B surface antigen. *J Biotechnol* **2006**, *121*, 471-481, doi:10.1016/j.jbiotec.2005.08.032.

23. Kalnciema, I.; Skrastina, D.; Ose, V.; Pumpens, P.; Zeltins, A. Potato virus Y-like particles as a new carrier for the presentation of foreign protein stretches. *Mol Biotechnol* **2012**, *52*, 129-139, doi:10.1007/s12033-011-9480-9.

24. Natilla, A.; Piazzolla, G.; Nuzzaci, M.; Saldarelli, P.; Tortorella, C.; Antonaci, S.; Piazzolla, P. Cucumber mosaic virus as carrier of a hepatitis C virus-derived epitope. *Arch Virol* **2004**, *149*, 137-154, doi:10.1007/s00705-003-0190-x.

25. Piazzolla, G.; Nuzzaci, M.; Tortorella, C.; Panella, E.; Natilla, A.; Boscia, D.; De Stradis, A.; Piazzolla, P.; Antonaci, S. Immunogenic properties of a chimeric plant virus expressing a hepatitis C virus (HCV)-derived epitope: new prospects for an HCV vaccine. *J Clin Immunol* **2005**, *25*, 142-152, doi:10.1007/s10875-005-2820-4.

26. Nuzzaci, M.; Piazzolla, G.; Vitti, A.; Lapelosa, M.; Tortorella, C.; Stella, I.; Natilla, A.; Antonaci, S.; Piazzolla, P. Cucumber mosaic virus as a presentation system for a double hepatitis C virus-derived epitope. *Arch Virol* **2007**, *152*, 915-928, doi:10.1007/s00705-006-0916-7.

27. Nuzzaci, M.; Bochicchio, I.; De Stradis, A.; Vitti, A.; Natilla, A.; Piazzolla, P.; Tamburro, A.M. Structural and biological properties of Cucumber mosaic virus particles carrying hepatitis C virus-derived epitopes. *J Virol Methods* **2009**, *155*, 118-121, doi:10.1016/j.jviromet.2008.10.005.

28. Nuzzaci, M.; Vitti, A.; Condelli, V.; Lanorte, M.T.; Tortorella, C.; Boscia, D.; Piazzolla, P.; Piazzolla, G. In vitro stability of Cucumber mosaic virus nanoparticles carrying a Hepatitis C virus-derived epitope under simulated gastrointestinal conditions and in vivo efficacy of an edible vaccine. *J Virol Methods* **2010**, *165*, 211-215, doi:10.1016/j.jviromet.2010.01.021.

29. Denis, J.; Majeau, N.; Acosta-Ramirez, E.; Savard, C.; Bedard, M.C.; Simard, S.; Lecours, K.; Bolduc, M.; Pare, C.; Willems, B., et al. Immunogenicity of papaya mosaic virus-like particles fused to a hepatitis C virus epitope: evidence for the critical function of multimerization. *Virology* **2007**, *363*, 59-68, doi:10.1016/j.virol.2007.01.011.

30. Uhde-Holzem, K.; Schlosser, V.; Viazov, S.; Fischer, R.; Commandeur, U. Immunogenic properties of chimeric potato virus X particles displaying the hepatitis C virus hypervariable region I peptide R9. *J Virol Methods* **2010**, *166*, 12-20, doi:10.1016/j.jviromet.2010.01.017.

31. McLain, L.; Porta, C.; Lomonossoff, G.P.; Durrani, Z.; Dimmock, N.J. Human immunodeficiency virus type 1-neutralizing antibodies raised to a glycoprotein 41 peptide expressed on the surface of a plant virus. *AIDS Res Hum Retroviruses* **1995**, *11*, 327-334, doi:10.1089/aid.1995.11.327.

32. McLain, L.; Durrani, Z.; Wisniewski, L.A.; Porta, C.; Lomonossoff, G.P.; Dimmock, N.J. Stimulation of neutralizing antibodies to human immunodeficiency virus type 1 in three strains of mice immunized with a 22 amino acid peptide of gp41 expressed on the surface of a plant virus. *Vaccine* **1996**, *14*, 799-810.

33. Durrani, Z.; McInerney, T.L.; McLain, L.; Jones, T.; Bellaby, T.; Brennan, F.R.; Dimmock, N.J. Intranasal immunization with a plant virus expressing a peptide from HIV-1 gp41 stimulates better mucosal and systemic HIV-1-specific IgA and IgG than oral immunization. *J Immunol Methods* **1998**, *220*, 93-103.

34. McInerney, T.L.; Brennan, F.R.; Jones, T.D.; Dimmock, N.J. Analysis of the ability of five adjuvants to enhance immune responses to a chimeric plant virus displaying an HIV-1 peptide. *Vaccine* **1999**, *17*, 1359-1368.

35. Joelson, T.; Akerblom, L.; Oxelfelt, P.; Strandberg, B.; Tomenius, K.; Morris, T.J. Presentation of a foreign peptide on the surface of tomato bushy stunt virus. *J Gen Virol* **1997**, *78 ( Pt 6)*, 1213-1217, doi:10.1099/0022-1317-78-6-1213.

36. Marusic, C.; Rizza, P.; Lattanzi, L.; Mancini, C.; Spada, M.; Belardelli, F.; Benvenuto, E.; Capone, I. Chimeric plant virus particles as immunogens for inducing murine and human immune responses against human immunodeficiency virus type 1. *J Virol* **2001**, *75*, 8434-8439.

37. Damodharan, S.; Gujar, R.; Pattabiraman, S.; Nesakumar, M.; Hanna, L.E.; Vadakkuppattu, R.D.; Usha, R. Expression and immunological characterization of cardamom mosaic virus coat protein displaying HIV gp41 epitopes. *Microbiol Immunol* **2013**, *57*, 374-385, doi:10.1111/1348-0421.12045.

38. Arcangeli, C.; Circelli, P.; Donini, M.; Aljabali, A.A.; Benvenuto, E.; Lomonossoff, G.P.; Marusic, C. Structure-based design and experimental engineering of a plant virus nanoparticle for the presentation of immunogenic epitopes and as a drug carrier. *J Biomol Struct Dyn* **2014**, *32*, 630-647, doi:10.1080/07391102.2013.785920.

39. Pokorna, D.; Cerovska, N.; Smahel, M.; Moravec, T.; Ludvikova, V.; Mackova, J.; Synkova, H.; Duskova, M.; Hozak, P.; Veleminsky, J. DNA vaccines based on chimeric potyvirus-like particles carrying HPV16 E7 peptide (aa 44-60). *Oncol Rep* **2005**, *14*, 1045-1053.

40. Plchova, H.; Moravec, T.; Hoffmeisterova, H.; Folwarczna, J.; Cerovska, N. Expression of Human papillomavirus 16 E7ggg oncoprotein on N- and C-terminus of Potato virus X coat protein in bacterial and plant cells. *Protein Expr Purif* **2011**, *77*, 146-152, doi:10.1016/j.pep.2011.01.008.

41. Cerovska, N.; Hoffmeisterova, H.; Moravec, T.; Plchova, H.; Folwarczna, J.; Synkova, H.; Ryslava, H.; Ludvikova, V.; Smahel, M. Transient expression of Human papillomavirus type 16 L2 epitope fused to N- and C-terminus of coat protein of Potato virus X in plants. *J Biosciences* **2012**, *37*, 125-133, doi:10.1007/s12038-011-9177-z.

42. Cerovska, N.; Moravec, T.; Hoffmeisterova, H.; Plchova, H.; Synkova, H.; Polakova, I.; Duskova, M.; Smahel, M. Expression of a recombinant Human papillomavirus 16 E6GT oncoprotein fused to N- and C-termini of Potato virus X coat protein in Nicotiana benthamiana. *Plant Cell Tiss Org* **2013**, *113*, 81-90, doi:10.1007/s11240-012-0253-3.

43. Vaculik, P.; Plchova, H.; Moravec, T.; Hoffmeisterova, H.; Cerovska, N.; Smahel, M. Potato virus X displaying the E7 peptide derived from human papillomavirus type 16: a novel position for epitope presentation. *Plant Cell Tiss Org* **2015**, *120*, 671-680, doi:10.1007/s11240-014-0634-x.

44. Porta, C.; Spall, V.E.; Loveland, J.; Johnson, J.E.; Barker, P.J.; Lomonossoff, G.P. Development of cowpea mosaic virus as a high-yielding system for the presentation of foreign peptides. *Virology* **1994**, *202*, 949-955, doi:10.1006/viro.1994.1417.

45. Porta, C.; Spall, V.E.; Lin, T.; Johnson, J.E.; Lomonossoff, G.P. The development of cowpea mosaic virus as a potential source of novel vaccines. *Intervirology* **1996**, *39*, 79-84.

46. Chen, T.H.; Chen, T.H.; Hu, C.C.; Liao, J.T.; Lee, C.W.; Liao, J.W.; Lin, M.Y.; Liu, H.J.; Wang, M.Y.; Lin, N.S., et al. Induction of protective immunity in chickens immunized with plant-made chimeric Bamboo mosaic virus particles expressing very virulent Infectious bursal disease virus antigen. *Virus Res* **2012**, *166*, 109-115, doi:10.1016/j.virusres.2012.02.021.

47. Sahithi, K.D.; Nancy, P.A.; Vishnu Vardhan, G.P.; Kumanan, K.; Vijayarani, K.; Hema, M. Detection of infectious bursal disease virus (IBDV) antibodies using chimeric plant virus-like particles. *Vet Microbiol* **2019**, *229*, 20-27, doi:10.1016/j.vetmic.2018.12.008.

48. Denis, J.; Acosta-Ramirez, E.; Zhao, Y.; Hamelin, M.E.; Koukavica, I.; Baz, M.; Abed, Y.; Savard, C.; Pare, C.; Lopez Macias, C., et al. Development of a universal influenza A vaccine based on the M2e peptide fused to the papaya mosaic virus (PapMV) vaccine platform. *Vaccine* **2008**, *26*, 3395-3403, doi:10.1016/j.vaccine.2008.04.052.

49. Leclerc, D.; Beauseigle, D.; Denis, J.; Morin, H.; Pare, C.; Lamarre, A.; Lapointe, R. Proteasome-independent major histocompatibility complex class I cross-presentation mediated by papaya mosaic virus-like particles leads to expansion of specific human T cells. *J Virol* **2007**, *81*, 1319-1326, doi:10.1128/JVI.01720-06.

50. Hanafi, L.A.; Bolduc, M.; Gagne, M.E.; Dufour, F.; Langelier, Y.; Boulassel, M.R.; Routy, J.P.; Leclerc, D.; Lapointe, R. Two distinct chimeric potexviruses share antigenic cross-presentation properties of MHC class I epitopes. *Vaccine* **2010**, *28*, 5617-5626, doi:10.1016/j.vaccine.2010.06.024.

51. Rioux, G.; Babin, C.; Majeau, N.; Leclerc, D. Engineering of papaya mosaic virus (PapMV) nanoparticles through fusion of the HA11 peptide to several putative surface-exposed sites. *PLoS One* **2012**, *7*, e31925, doi:10.1371/journal.pone.0031925.

52. Babin, C.; Majeau, N.; Leclerc, D. Engineering of papaya mosaic virus (PapMV) nanoparticles with a CTL epitope derived from influenza NP. *J Nanobiotechnology* **2013**, *11*, 10, doi:10.1186/1477-3155-11-10.

53. Carignan, D.; Therien, A.; Rioux, G.; Paquet, G.; Gagne, M.E.; Bolduc, M.; Savard, P.; Leclerc, D. Engineering of the PapMV vaccine platform with a shortened M2e peptide leads to an effective one dose influenza vaccine. *Vaccine* **2015**, *33*, 7245-7253, doi:10.1016/j.vaccine.2015.10.123.

54. Therien, A.; Bedard, M.; Carignan, D.; Rioux, G.; Gauthier-Landry, L.; Laliberte-Gagne, M.E.; Bolduc, M.; Savard, P.; Leclerc, D. A versatile papaya mosaic virus (PapMV) vaccine platform based on sortase-mediated antigen coupling. *J Nanobiotechnology* **2017**, *15*, 54, doi:10.1186/s12951-017-0289-y.

55. Bolduc, M.; Baz, M.; Laliberte-Gagne, M.E.; Carignan, D.; Garneau, C.; Russel, A.; Boivin, G.; Savard, P.; Leclerc, D. The quest for a nanoparticle-based vaccine inducing broad protection to influenza viruses. *Nanomedicine* **2018**, *14*, 2563-2574, doi:10.1016/j.nano.2018.08.010.

56. Laliberte-Gagne, M.E.; Bolduc, M.; Therien, A.; Garneau, C.; Casault, P.; Savard, P.; Estaquier, J.; Leclerc, D. Increased Immunogenicity of Full-Length Protein Antigens through Sortase-Mediated Coupling on the PapMV Vaccine Platform. *Vaccines (Basel)* **2019**, *7*, doi:10.3390/vaccines7020049.

57. Lico, C.; Mancini, C.; Italiani, P.; Betti, C.; Boraschi, D.; Benvenuto, E.; Baschieri, S. Plant-produced potato virus X chimeric particles displaying an influenza virus-derived peptide activate specific CD8+ T cells in mice. *Vaccine* **2009**, *27*, 5069-5076, doi:10.1016/j.vaccine.2009.06.045.

58. Tyulkina, L.G.; Skurat, E.V.; Frolova, O.Y.; Komarova, T.V.; Karger, E.M.; Atabekov, I.G. New viral vector for superproduction of epitopes of vaccine proteins in plants. *Acta Naturae* **2011**, *3*, 73-82.

59. Hassani-Mehraban, A.; Creutzburg, S.; van Heereveld, L.; Kormelink, R. Feasibility of Cowpea chlorotic mottle virus-like particles as scaffold for epitope presentations. *BMC Biotechnol* **2015**, *15*, 80, doi:10.1186/s12896-015-0180-6.

60. Cantin, G.T.; Resnick, S.; Jin, H.F.; O'Hanlon, R.; Espinosa, O.; Stevens, A.; Payne, J.; Glenn, N.R.; Rasochova, L.; Allen, J.R. Comparison of Methods for Chemical Conjugation of an Influenza Peptide to Wild-Type and Cysteine-Mutant Virus-Like Particles Expressed in Pseudomonas fluorescens. *Int J Pept Res Ther* **2011**, *17*, 217-224, doi:10.1007/s10989-011-9259-7.

61. Leclerc, D.; Rivest, M.; Babin, C.; Lopez-Macias, C.; Savard, P. A novel M2e based flu vaccine formulation for dogs. *PLoS One* **2013**, *8*, e77084, doi:10.1371/journal.pone.0077084.

62. Mallajosyula, J.K.; Hiatt, E.; Hume, S.; Johnson, A.; Jeevan, T.; Chikwamba, R.; Pogue, G.P.; Bratcher, B.; Haydon, H.; Webby, R.J., et al. Single-dose monomeric HA subunit vaccine generates full protection from influenza challenge. *Hum Vaccin Immunother* **2014**, *10*, 586-595.

63. Cardenas-Vargas, A.; Elizondo-Quiroga, D.; Gutierrez-Ortega, A.; Charles-Nino, C.; Pedroza-Roldan, C. Evaluation of the Immunogenicity of a Potyvirus-Like Particle as an Adjuvant of a Synthetic Peptide. *Viral Immunol* **2016**, *29*, 557-564, doi:10.1089/vim.2016.0087.

64. Arnaboldi, P.M.; Sambir, M.; D'Arco, C.; Peters, L.A.; Seegers, J.F.; Mayer, L.; McCormick, A.A.; Dattwyler, R.J. Intranasal delivery of a protein subunit vaccine using a Tobacco Mosaic Virus platform protects against pneumonic plague. *Vaccine* **2016**, *34*, 5768-5776, doi:10.1016/j.vaccine.2016.09.063.

65. Shahana, P.V.; Das, D.; Gontu, A.; Chandran, D.; Maithal, K. Efficient production of Tymovirus like particles displaying immunodominant epitopes of Japanese Encephalitis Virus envelope protein. *Protein Expr Purif* **2015**, *113*, 35-43, doi:10.1016/j.pep.2015.03.017.

66. Chen, T.H.; Hu, C.C.; Liao, J.T.; Lee, Y.L.; Huang, Y.W.; Lin, N.S.; Lin, Y.L.; Hsu, Y.H. Production of Japanese Encephalitis Virus Antigens in Plants Using Bamboo Mosaic Virus-Based Vector. *Front Microbiol* **2017**, *8*, 788, doi:10.3389/fmicb.2017.00788.

67. Lacasse, P.; Denis, J.; Lapointe, R.; Leclerc, D.; Lamarre, A. Novel plant virus-based vaccine induces protective cytotoxic T-lymphocyte-mediated antiviral immunity through dendritic cell maturation. *J Virol* **2008**, *82*, 785-794, doi:10.1128/JVI.01811-07.

68. Turpen, T.H.; Reinl, S.J.; Charoenvit, Y.; Hoffman, S.L.; Fallarme, V.; Grill, L.K. Malarial epitopes expressed on the surface of recombinant tobacco mosaic virus. *Biotechnology (N Y)* **1995**, *13*, 53-57.

69. Jones, R.M.; Chichester, J.A.; Mett, V.; Jaje, J.; Tottey, S.; Manceva, S.; Casta, L.J.; Gibbs, S.K.; Musiychuk, K.; Shamloul, M., et al. A plant-produced Pfs25 VLP malaria vaccine candidate induces persistent transmission blocking antibodies against Plasmodium falciparum in immunized mice. *PLoS One* **2013**, *8*, e79538, doi:10.1371/journal.pone.0079538.

70. Chichester, J.A.; Green, B.J.; Jones, R.M.; Shoji, Y.; Miura, K.; Long, C.A.; Lee, C.K.; Ockenhouse, C.F.; Morin, M.J.; Streatfield, S.J., et al. Safety and immunogenicity of a plant-produced Pfs25 virus-like particle as a transmission blocking vaccine against malaria: A Phase 1 dose-escalation study in healthy adults. *Vaccine* **2018**, *36*, 5865-5871, doi:10.1016/j.vaccine.2018.08.033.

71. Cabral-Miranda, G.; Heath, M.D.; Mohsen, M.O.; Gomes, A.C.; Engeroff, P.; Flaxman, A.; Leoratti, F.M.S.; El-Turabi, A.; Reyes-Sandoval, A.; Skinner, M.A., et al. Virus-Like Particle (VLP) Plus Microcrystalline Tyrosine (MCT) Adjuvants Enhance Vaccine Efficacy Improving T and B Cell Immunogenicity and Protection against Plasmodium berghei/vivax. *Vaccines (Basel)* **2017**, *5*, doi:10.3390/vaccines5020010.

72. Dalsgaard, K.; Uttenthal, A.; Jones, T.D.; Xu, F.; Merryweather, A.; Hamilton, W.D.; Langeveld, J.P.; Boshuizen, R.S.; Kamstrup, S.; Lomonossoff, G.P., et al. Plant-derived vaccine protects target animals against a viral disease. *Nat Biotechnol* **1997**, *15*, 248-252, doi:10.1038/nbt0397-248.

73. Koo, M.; Bendahmane, M.; Lettieri, G.A.; Paoletti, A.D.; Lane, T.E.; Fitchen, J.H.; Buchmeier, M.J.; Beachy, R.N. Protective immunity against murine hepatitis virus (MHV) induced by intranasal or subcutaneous administration of hybrids of tobacco mosaic virus that carries an MHV epitope. *P Natl Acad Sci USA* **1999**, *96*, 7774-7779, doi:DOI 10.1073/pnas.96.14.7774.

74. Zhao, Y.; Hammond, R.W. Development of a candidate vaccine for Newcastle disease virus by epitope display in the Cucumber mosaic virus capsid protein. *Biotechnol Lett* **2005**, *27*, 375-382, doi:10.1007/s10529-005-1773-2.

75. Natilla, A.; Hammond, R.W.; Nemchinov, L.G. Epitope presentation system based on cucumber mosaic virus coat protein expressed from a potato virus X-based vector. *Arch Virol* **2006**, *151*, 1373-1386, doi:10.1007/s00705-005-0711-x.

76. Natilla, A.; Nemchinov, L.G. Improvement of PVX/CMV CP expression tool for display of short foreign antigens. *Protein Expr Purif* **2008**, *59*, 117-121, doi:10.1016/j.pep.2008.01.011.

77. Haynes, J.R.; Cunningham, J.; von Seefried, A.; Lennick, M.; Garvin, R.T.; Shen, S.-H. Development of a Genetically-Engineered, Candidate Polio Vaccine Employing the Self-Assembling Properties of the Tobacco Mosaic Virus Coat Protein. *Nat Biotech* **1986**, *4*, 637-641, doi:10.1038/nbt0786-637.

78. Fujiyama, K.; Saejung, W.; Yanagihara, I.; Nakado, J.; Misaki, R.; Honda, T.; Watanabe, Y.; Seki, T. In Planta production of immunogenic poliovirus peptide using tobacco mosaic virus-based vector system. *J Biosci Bioeng* **2006**, *101*, 398-402, doi:10.1263/jbb.101.398.

79. Aguilera, B.E.; Chavez-Calvillo, G.; Elizondo-Quiroga, D.; Jimenez-Garcia, M.N.; Carrillo-Tripp, M.; Silva-Rosales, L.; Hernandez-Gutierrez, R.; Gutierrez-Ortega, A. Porcine circovirus type 2 protective epitope densely carried by chimeric papaya ringspot virus-like particles expressed in Escherichia coli as a cost-effective vaccine manufacture alternative. *Biotechnol Appl Biochem* **2017**, *64*, 406-414, doi:10.1002/bab.1491.

80. Gellert, A.; Salanki, K.; Tombacz, K.; Tuboly, T.; Balazs, E. A cucumber mosaic virus based expression system for the production of porcine circovirus specific vaccines. *PLoS One* **2012**, *7*, e52688, doi:10.1371/journal.pone.0052688.

81. Tran, H.H.; Chen, B.; Chen, H.; Menassa, R.; Hao, X.; Bernards, M.; Huner, N.P.A.; Wang, A. Development of a cucumber green mottle mosaic virus-based expression vector for the production in cucumber of neutralizing epitopes against a devastating animal virus. *J Virol Methods* **2019**, *269*, 18-25, doi:10.1016/j.jviromet.2019.04.006.

82. Brennan, F.R.; Gilleland, L.B.; Staczek, J.; Bendig, M.M.; Hamilton, W.D.; Gilleland, H.E., Jr. A chimaeric plant virus vaccine protects mice against a bacterial infection. *Microbiology* **1999**, *145 ( Pt 8)*, 2061-2067, doi:10.1099/13500872-145-8-2061.

83. Brennan, F.R.; Jones, T.D.; Gilleland, L.B.; Bellaby, T.; Xu, F.; North, P.C.; Thompson, A.; Staczek, J.; Lin, T.; Johnson, J.E., et al. Pseudomonas aeruginosa outer-membrane protein F epitopes are highly immunogenic in mice when expressed on a plant virus. *Microbiology* **1999**, *145 ( Pt 1)*, 211-220, doi:10.1099/13500872-145-1-211.

84. Gilleland, H.E.; Gilleland, L.B.; Staczek, J.; Harty, R.N.; Garcia-Sastre, A.; Palese, P.; Brennan, F.R.; Hamilton, W.D.; Bendahmane, M.; Beachy, R.N. Chimeric animal and plant viruses expressing epitopes of outer membrane protein F as a combined vaccine against Pseudomonas aeruginosa lung infection. *FEMS Immunol Med Microbiol* **2000**, *27*, 291-297.

85. Staczek, J.; Bendahmane, M.; Gilleland, L.B.; Beachy, R.N.; Gilleland, H.E., Jr. Immunization with a chimeric tobacco mosaic virus containing an epitope of outer membrane protein F of Pseudomonas aeruginosa provides protection against challenge with P. aeruginosa. *Vaccine* **2000**, *18*, 2266-2274.

86. Yusibov, V.; Modelska, A.; Steplewski, K.; Agadjanyan, M.; Weiner, D.; Hooper, D.C.; Koprowski, H. Antigens produced in plants by infection with chimeric plant viruses immunize against rabies virus and HIV-1. *Proc Natl Acad Sci U S A* **1997**, *94*, 5784-5788.

87. Yusibov, V.; Hooper, D.C.; Spitsin, S.V.; Fleysh, N.; Kean, R.B.; Mikheeva, T.; Deka, D.; Karasev, A.; Cox, S.; Randall, J., et al. Expression in plants and immunogenicity of plant virus-based experimental rabies vaccine. *Vaccine* **2002**, *20*, 3155-3164.

88. Nikitin, N.A.; Matveeva, I.N.; Trifonova, E.A.; Puhova, N.M.; Samuylenko, A.Y.; Gryn, S.A.; Atabekov, J.G.; Karpova, O.V. Spherical particles derived from TMV virions enhance the protective properties of the rabies vaccine. *Data Brief* **2018**, *21*, 742-745, doi:10.1016/j.dib.2018.10.030.

89. Ortega-Berlanga, B.; Musiychuk, K.; Shoji, Y.; Chichester, J.A.; Yusibov, V.; Patino-Rodriguez, O.; Noyola, D.E.; Alpuche-Solis, A.G. Engineering and expression of a RhoA peptide against respiratory syncytial virus infection in plants. *Planta* **2016**, *243*, 451-458, doi:10.1007/s00425-015-2416-z.

90. Kumar, S.; Ochoa, W.; Singh, P.; Hsu, C.; Schneemann, A.; Manchester, M.; Olson, M.; Reddy, V. Tomato bushy stunt virus (TBSV), a versatile platform for polyvalent display of antigenic epitopes and vaccine design. *Virology* **2009**, *388*, 185-190, doi:10.1016/j.virol.2009.02.051.

91. Trifonova, E.A.; Zenin, V.A.; Nikitin, N.A.; Yurkova, M.S.; Ryabchevskaya, E.M.; Putlyaev, E.V.; Donchenko, E.K.; Kondakova, O.A.; Fedorov, A.N.; Atabekov, J.G., et al. Study of rubella candidate vaccine based on a structurally modified plant virus. *Antiviral Res* **2017**, *144*, 27-33, doi:10.1016/j.antiviral.2017.05.006.

92. Brennan, F.R.; Bellaby, T.; Helliwell, S.M.; Jones, T.D.; Kamstrup, S.; Dalsgaard, K.; Flock, J.I.; Hamilton, W.D. Chimeric plant virus particles administered nasally or orally induce systemic and mucosal immune responses in mice. *J Virol* **1999**, *73*, 930-938.

93. Pomwised, R.; Intamaso, U.; Teintze, M.; Young, M.; Pincus, S.H. Coupling Peptide Antigens to Virus-Like Particles or to Protein Carriers Influences the Th1/Th2 Polarity of the Resulting Immune Response. *Vaccines (Basel)* **2016**, *4*, doi:10.3390/vaccines4020015.

94. Cabral-Miranda, G.; Lim, S.M.; Mohsen, M.O.; Pobelov, I.V.; Roesti, E.S.; Heath, M.D.; Skinner, M.A.; Kramer, M.E.; Martina, B.E.E.; Bachmann, M.F. Zika Virus-Derived E-DIII Protein Displayed on Immunologically Optimized VLPs Induces Neutralizing Antibodies without Causing Enhancement of Dengue Virus Infection. *Vaccines-Basel* **2019**, *7*, doi:ARTN 72 10.3390/vaccines7030072.
