# Supplementary material for: Recent Advances in the Use of Plant Virus-Like Particles as Vaccines
Source: Viruses. 2020 Feb 28;12(3):270. doi: 10.3390/v12030270 (PMC7150928; doi:10.3390/v12030270)
Supplement: Supplementary file 1 [file viruses-12-00270-s001.zip › Suppl_Table_3_anticancer_vaccines.docx]

Supplement Table 3. Examples of vaccine candidates against cancers based on plant virus carriers (updated from Balke I., Zeltins A. (2019) Adv.Drug Deliv. Rev. 145:119-129)

| **Vaccine candidate** | **Antigen** | **Plant virus / VLP** | **Expression system** | **Immunological data** | **Reference** |
| --- | --- | --- | --- | --- | --- |

B-cell Idiotypic (Id) antigen from IgM / BCL1 cells PVX Plant (virus) Id-VLPs induce strong antibody response [1]

lymphoma conjugated Mammalian with dominating IgG2a subtype

antigen (antigen)

Breast cancer HER2/neu TMV Plant Modified peptide-VLP fusion binds to [2]

E75 peptide (AA 369-377) specific mAb (trastuzumab)

HER2 peptides PVX Plant Peptide-VLPs elicit the production of [3,4]

CH401 (AA 163-182), P4 (AA 378-394) HER2-specific antibodies in mice

HER2 peptides CPMV Plant Peptide-VLPs elicit the production of [4]

CH401 (AA 163-182), P4 (AA 378-394) HER2-specific antibodies in mice

HER2 CH401 epitope CCMV Plant *In vivo* tumor challenge of preimmunized [5]

CPMV mice reduces tumor growth and improved

SeMV survival

No introduced antigen CPMV Plant CPMV with chemotherapy reduces tumor [6]

growth and suppresses distant tumor

development

Melanoma Melanoma-associated peptides (MART, PVX Plant data not found [7]

MARTSh, NY-ESO, NY-ESOSh)

Melanoma peptide p15 TMV Plant p15-VLPs improve the survival in mouse [8,9]

B16model of melanoma

| **Vaccine candidate** | **Antigen** | **Plant virus / VLP** | **Expression system** | **Immunological data** | **Reference** |
| --- | --- | --- | --- | --- | --- |

Melanoma Melanoma gp100 peptide PapMV *E.coli* Plant VLPs mediate MHC class I cross- [10,11]

presentation and induce T-cell proliferation

Melanoma gp100 peptide MaMV *E.coli* VLPs induce T-cell proliferation [11]

no introduced antigen CPMV Plant CPMV particles reduce the tumor growth [12]

TMV more efficiently than TMV

LCMV p33 peptide (9 AA) CMV *E.coli* CMV-p33 vaccine formulated with micro- [13]

conjugated crystalline tyrosine enhances the specific

peptide T cell response and reduces the tumor

volume in mice

Malignant no introduced antigen CPMV Plant Empty CPMV particle immunotherapy [14]

glioma leads to brain tumor regression in mice

Ovarian cancer no introduced antigen CPMV Plant Combined treatment using plant VLPs and [15]

radiation therapy reduces tumor growth

no introduced antigen CPMV Plant CPMV-polyamidoamine dendrimer [16]

aggregates reduce the tumor progression

in mice

no introduced antigen CPMV Plant Empty and RNA-containing CPMV promotes [17]

Insect cells tumor regression and boosts populations of

infiltrating neutrophils and dendritic cells

**References**

1. Jobsri, J.; Allen, A.; Rajagopal, D.; Shipton, M.; Kanyuka, K.; Lomonossoff, G.P.; Ottensmeier, C.; Diebold, S.S.; Stevenson, F.K.; Savelyeva, N. Plant virus particles carrying tumour antigen activate TLR7 and Induce high levels of protective antibody. *PLoS One* **2015**, *10*, e0118096, doi:10.1371/journal.pone.0118096.

2. Frolova, O.Y.; Petrunia, I.V.; Komarova, T.V.; Kosorukov, V.S.; Sheval, E.V.; Gleba, Y.Y.; Dorokhov, Y.L. Trastuzumab-binding peptide display by Tobacco mosaic virus. *Virology* **2010**, *407*, 7-13, doi:10.1016/j.virol.2010.08.005.

3. Shukla, S.; Wen, A.M.; Commandeur, U.; Steinmetz, N.F. Presentation of HER2 epitopes using a filamentous plant virus-based vaccination platform. *J Mater Chem B* **2014**, *2*, 6249-6258, doi:10.1039/c4tb00749b.

4. Shukla, S.; Myers, J.T.; Woods, S.E.; Gong, X.; Czapar, A.E.; Commandeur, U.; Huang, A.Y.; Levine, A.D.; Steinmetz, N.F. Plant viral nanoparticles-based HER2 vaccine: Immune response influenced by differential transport, localization and cellular interactions of particulate carriers. *Biomaterials* **2017**, *121*, 15-27, doi:10.1016/j.biomaterials.2016.12.030.

5. Cai, H.; Shukla, S.; Wang, C.; Masarapu, H.; Steinmetz, N.F. Heterologous Prime-Boost Enhances the Antitumor Immune Response Elicited by Plant-Virus-Based Cancer Vaccine. *J Am Chem Soc* **2019**, *141*, 6509-6518, doi:10.1021/jacs.9b01523.

6. Cai, H.; Wang, C.; Shukla, S.; Steinmetz, N.F. Cowpea Mosaic Virus Immunotherapy Combined with Cyclophosphamide Reduces Breast Cancer Tumor Burden and Inhibits Lung Metastasis. *Adv Sci (Weinh)* **2019**, *6*, 1802281, doi:10.1002/advs.201802281.

7. Lico, C.; Capuano, F.; Renzone, G.; Donini, M.; Marusic, C.; Scaloni, A.; Benvenuto, E.; Baschieri, S. Peptide display on Potato virus X: molecular features of the coat protein-fused peptide affecting cell-to-cell and phloem movement of chimeric virus particles. *J Gen Virol* **2006**, *87*, 3103-3112, doi:10.1099/vir.0.82097-0.

8. McCormick, A.A.; Corbo, T.A.; Wykoff-Clary, S.; Nguyen, L.V.; Smith, M.L.; Palmer, K.E.; Pogue, G.P. TMV-peptide fusion vaccines induce cell-mediated immune responses and tumor protection in two murine models. *Vaccine* **2006**, *24*, 6414-6423, doi:10.1016/j.vaccine.2006.06.003.

9. McCormick, A.A.; Corbo, T.A.; Wykoff-Clary, S.; Palmer, K.E.; Pogue, G.P. Chemical conjugate TMV-peptide bivalent fusion vaccines improve cellular immunity and tumor protection. *Bioconjug Chem* **2006**, *17*, 1330-1338, doi:10.1021/bc060124m.

10. Leclerc, D.; Beauseigle, D.; Denis, J.; Morin, H.; Pare, C.; Lamarre, A.; Lapointe, R. Proteasome-independent major histocompatibility complex class I cross-presentation mediated by papaya mosaic virus-like particles leads to expansion of specific human T cells. *J Virol* **2007**, *81*, 1319-1326, doi:10.1128/JVI.01720-06.

11. Hanafi, L.A.; Bolduc, M.; Gagne, M.E.; Dufour, F.; Langelier, Y.; Boulassel, M.R.; Routy, J.P.; Leclerc, D.; Lapointe, R. Two distinct chimeric potexviruses share antigenic cross-presentation properties of MHC class I epitopes. *Vaccine* **2010**, *28*, 5617-5626, doi:10.1016/j.vaccine.2010.06.024.

12. Murray, A.A.; Wang, C.; Fiering, S.; Steinmetz, N.F. In Situ Vaccination with Cowpea vs Tobacco Mosaic Virus against Melanoma. *Mol Pharm* **2018**, *15*, 3700-3716, doi:10.1021/acs.molpharmaceut.8b00316.

13. Mohsen, M.O.; Heath, M.D.; Cabral-Miranda, G.; Lipp, C.; Zeltins, A.; Sande, M.; Stein, J.V.; Riether, C.; Roesti, E.; Zha, L.S., et al. Vaccination with nanoparticles combined with micro-adjuvants protects against cancer. *J Immunother Cancer* **2019**, *7*, doi:ARTN 114 10.1186/s40425-019-0587-z.

14. Kerstetter-Fogle, A.; Shukla, S.; Wang, C.; Beiss, V.; Harris, P.L.R.; Sloan, A.E.; Steinmetz, N.F. Plant Virus-Like Particle In Situ Vaccine for Intracranial Glioma Immunotherapy. *Cancers (Basel)* **2019**, *11*, doi:10.3390/cancers11040515.

15. Patel, R.; Czapar, A.E.; Fiering, S.; Oleinick, N.L.; Steinmetz, N.F. Radiation Therapy Combined with Cowpea Mosaic Virus Nanoparticle in Situ Vaccination Initiates Immune-Mediated Tumor Regression. *ACS Omega* **2018**, *3*, 3702-3707, doi:10.1021/acsomega.8b00227.

16. Czapar, A.E.; Tiu, B.D.B.; Veliz, F.A.; Pokorski, J.K.; Steinmetz, N.F. Slow-Release Formulation of Cowpea Mosaic Virus for In Situ Vaccine Delivery to Treat Ovarian Cancer. *Adv Sci (Weinh)* **2018**, *5*, 1700991, doi:10.1002/advs.201700991.

17. Wang, C.; Beiss, V.; Steinmetz, N.F. Cowpea Mosaic Virus Nanoparticles and Empty Virus-Like Particles Show Distinct but Overlapping Immunostimulatory Properties. *J Virol* **2019**, *93*, doi:10.1128/JVI.00129-19.
