# Supplementary material for: Recent Advances in the Use of Plant Virus-Like Particles as Vaccines
Source: Viruses. 2020 Feb 28;12(3):270. doi: 10.3390/v12030270 (PMC7150928; doi:10.3390/v12030270)
Supplement: Supplementary file 1 [file viruses-12-00270-s001.zip › Suppl_Table_4_allergy_autoimmune_vaccines.docx]

Supplement Table 3. Examples of vaccine candidates against allergies, autoimmune and other diseases based on plant virus carriers.

| **Vaccine candidate** | **Antigen** | **Plant virus / VLP** | **Expression system** | **Immunological data** | **Reference** |
| --- | --- | --- | --- | --- | --- |

Alzheimer’s disease amyloid β N-terminal peptides (AA 1-15) CMV Plant Polyclonal antibodies against Aβ [1]

recognize VLPs

amyloid β N-terminal peptide (AA 1-6) CMV *E. coli* Antibodies from immunized mice [2]

recognize human Alzheimer plaques

Atopic dermatitis Canine IL31 CMV *E.coli* Vaccinated dogs demonstrate reduced [3]

symptoms of atopic dermatitis

Cat allergy Major cat allergen Fel_d_1 CMV *E.coli* Immunized mice are protected against [2]

systemic anaphylactic reactions

Major cat allergen Fel_d_1 CMV *E.coli* Vaccinated cats have decreased level [4]

of endogenous Fel_d_1; reduced risk of

allergic symptoms for cat owners

Insect bite Horse IL5 CMV *E.coli* Vaccinated horses demonstrate [5,6]

hypersensitivity reduced symptoms of insect bite

hypersensitivity

Horse IL31 CMV *E.coli* Vaccinated horses demonstrate reduced [7]

symptoms of insect bite

hypersensitivity

Pain Mice NGF CMV *E.coli* (VLPs) Reduction of spontaneous pain behaviour [8]

Mammalian in vaccinated mice with surgically induced

(NGF) osteoartritis

Peanut allergy allergen Ara_h_202 CMV *E.coli* Vaccinated mice protected against [9]

systemic anaphylactic reactions

Psoriasis Mice IL17A CMV *E.coli* Immunized mice demonstrate the [2]

reduction of psoriatic symptoms

**References**

1. Vitti, A.; Piazzolla, G.; Condelli, V.; Nuzzaci, M.; Lanorte, M.T.; Boscia, D.; De Stradis, A.; Antonaci, S.; Piazzolla, P.; Tortorella, C. Cucumber mosaic virus as the expression system for a potential vaccine against Alzheimer's disease. *J Virol Methods* **2010**, *169*, 332-340, doi:10.1016/j.jviromet.2010.07.039.

2. Zeltins, A.; West, J.; Zabel, F.; El Turabi, A.; Balke, I.; Haas, S.; Maudrich, M.; Storni, F.; Engeroff, P.; Jennings, G.T., et al. Incorporation of tetanus-epitope into virus-like particles achieves vaccine responses even in older recipients in models of psoriasis, Alzheimer's and cat allergy. *NPJ Vaccines* **2017**, *2*, 30, doi:10.1038/s41541-017-0030-8.

3. Bachmann, M.F.; Zeltins, A.; Kalnins, G.; Balke, I.; Fischer, N.; Rostaher, A.; Tars, K.; Favrot, C. Vaccination against IL-31 for the treatment of atopic dermatitis in dogs. *J Allergy Clin Immunol* **2018**, *142*, 279-281 e271, doi:10.1016/j.jaci.2017.12.994.

4. Thoms, F.; Jennings, G.T.; Maudrich, M.; Vogel, M.; Haas, S.; Zeltins, A.; Hofmann-Lehmann, R.; Riond, B.; Grossmann, J.; Hunziker, P., et al. Immunization of cats to induce neutralizing antibodies against Fel d 1, the major feline allergen in human subjects. *J Allergy Clin Immunol* **2019**, *144*, 193-203, doi:10.1016/j.jaci.2019.01.050.

5. Fettelschoss-Gabriel, A.; Fettelschoss, V.; Thoms, F.; Giese, C.; Daniel, M.; Olomski, F.; Kamarachev, J.; Birkmann, K.; Buhler, M.; Kummer, M., et al. Treating insect-bite hypersensitivity in horses with active vaccination against IL-5. *J Allergy Clin Immunol* **2018**, *142*, 1194-1205 e1193, doi:10.1016/j.jaci.2018.01.041.

6. Fettelschoss-Gabriel, A.; Fettelschoss, V.; Olomski, F.; Birkmann, K.; Thoms, F.; Buhler, M.; Kummer, M.; Zeltins, A.; Kundig, T.M.; Bachmann, M.F. Active vaccination against interleukin-5 as long-term treatment for insect-bite hypersensitivity in horses. *Allergy* **2019**, *74*, 572-582, doi:10.1111/all.13659.

7. Olomski, F.; Fettelschoss, V.; Jonsdottir, S.; Birkmann, K.; Thoms, F.; Marti, E.; Bachmann, M.F.; Kundig, T.M.; Fettelschoss-Gabriel, A. Interleukin 31 in insect bite hypersensitivity - Alleviating clinical symptoms by active vaccination against itch. *Allergy* **2019**, 10.1111/all.14145, doi:10.1111/all.14145.

8. von Loga, I.S.; El-Turabi, A.; Jostins, L.; Miotla-Zarebska, J.; Mackay-Alderson, J.; Zeltins, A.; Parisi, I.; Bachmann, M.F.; Vincent, T.L. Active immunisation targeting nerve growth factor attenuates chronic pain behaviour in murine osteoarthritis. *Ann Rheum Dis* **2019**, *78*, 672-675, doi:10.1136/annrheumdis-2018-214489.

9. Storni, F.; Zeltins, A.; Balke, I.; Heath, M.D.; Kramer, M.F.; Skinner, M.A.; Zha, L.; Roesti, E.; Engeroff, P.; Muri, L., et al. Vaccine against peanut allergy based on engineered Virus-Like-Particles displaying single major peanut allergens. *J Allergy Clin Immunol* **2019**, 10.1016/j.jaci.2019.12.007, doi:10.1016/j.jaci.2019.12.007.
